# A customized nuclear target enrichment approach for developing a phylogenomic baseline for *Dioscorea* yams (Dioscoreaceae)

**DOI:** 10.1002/aps3.11254

**Published:** 2019-06-13

**Authors:** Marybel Soto Gomez, Lisa Pokorny, Michael B. Kantar, Félix Forest, Ilia J. Leitch, Barbara Gravendeel, Paul Wilkin, Sean W. Graham, Juan Viruel

**Affiliations:** ^1^ Department of Botany University of British Columbia 6270 University Boulevard Vancouver British Columbia V6T 1Z4 Canada; ^2^ UBC Botanical Garden and Centre for Plant Research University of British Columbia 6804 Marine Drive SW Vancouver British Columbia V6T 1Z4 Canada; ^3^ Royal Botanic Gardens Kew, Richmond Surrey TW9 3DS United Kingdom; ^4^ Department of Tropical Plant and Soil Sciences University of Hawai‘i at Manoa Honolulu Hawai‘i 96822 USA; ^5^ Naturalis Biodiversity Center Endless Forms Sylviusweg 72 Leiden 2333 BE The Netherlands; ^6^ Institute Biology Leiden Leiden University Sylviusweg 72 Leiden 2333 BE The Netherlands; ^7^ Faculty of Science and Technology University of Applied Sciences Leiden Zernikedreef 11 Leiden 2333 CK The Netherlands

**Keywords:** *Dioscorea* target capture kit, herbarium, Hyb‐Seq, monocot phylogenomics, ortholog identification, tuber crop wild relatives

## Abstract

**Premise:**

We developed a target enrichment panel for phylogenomic studies of *Dioscorea*, an economically important genus with incompletely resolved relationships.

**Methods:**

Our bait panel comprises 260 low‐ to single‐copy nuclear genes targeted to work in *Dioscorea*, assessed here using a preliminary taxon sampling that includes both distantly and closely related taxa, including several yam crops and potential crop wild relatives. We applied coalescent‐based and maximum likelihood phylogenomic inference approaches to the pilot taxon set, incorporating new and published transcriptome data from additional species.

**Results:**

The custom panel retrieved ~94% of targets and >80% of full gene length from 88% and 68% of samples, respectively. It has minimal gene overlap with existing panels designed for angiosperm‐wide studies and generally recovers longer and more variable targets. Pilot phylogenomic analyses consistently resolve most deep and recent relationships with strong support across analyses and point to revised relationships between the crop species *D. alata* and candidate crop wild relatives.

**Discussion:**

Our customized panel reliably retrieves targeted loci from *Dioscorea*, is informative for resolving relationships in denser samplings, and is suitable for refining our understanding of the independent origins of cultivated yam species; the panel likely has broader promise for phylogenomic studies across Dioscoreales.

The *Dioscorea* L. yams (Dioscoreaceae) are a genus of ~625 species (Govaerts et al., 2018) that have highly diverse underground storage organs. Around nine species with starchy, annually replaced tubers are cultivated on a large scale in ~50 countries (Lebot, 2009; Asiedu and Sartie, 2010; Bhattacharjee et al., 2011); two of these, *D. alata* L. and *D. cayenensis* Lam., are a primary staple food for over 100 million people, predominantly in Western Africa (Mignouna et al., 2003; note that the cultivated form of *D. cayenensis* is sometimes referred to as *D. cayenensis* subsp. *rotundata* (Poir.) J. Miège, or as *D. rotundata* Poir.). An additional ~40–50 species are cultivated or wild‐harvested as food (Martin and Degras, 1978). Yam lineages with perennial tubers and rhizomes have also been used as a main source of steroidal precursors (De Luca et al., 2012; Hua et al., 2017) and traditional medicines (Lebot, 2009). Our understanding of *Dioscorea* evolution has benefited greatly from previous studies using two to six plastid markers (Wilkin et al., 2005; Hsu et al., 2013; Maurin et al., 2016; Viruel et al., 2016; Couto et al., 2018) or a single nuclear gene (Viruel et al., 2018); these studies provide a baseline phylogenetic outline for the genus. However, relationships among most major clades and species remain highly uncertain, in part due to relatively limited taxon and gene sampling, coupled with low levels of molecular variation in sampled markers. For example, multiple poorly supported internal branches were associated with short branch lengths in Wilkin et al. (2005), who suggested that the two plastid markers they used were not sufficiently informative for inferring those relationships. The studies with the densest sampling to date included representatives of the approximately 10 major *Dioscorea* clades (Wilkin et al., 2005; Viruel et al., 2016, 2018; Couto et al., 2018), but only 8–22% of the species in the genus. Sampling of crops and possible crop wild relatives (CWRs) has also remained relatively limited (e.g., Terauchi et al., 1992; Malapa et al., 2005; Mignouna et al., 2005; Mukherjee and Bhat, 2013; Girma et al., 2014, 2016; Chaïr et al., 2016), and their inter‐relationships have yet to be resolved (e.g., confirming the identity of CWRs of *D. alata*; Chaïr et al., 2016). Here we present and apply methodology to enable phylogenomic research in yams, based on hundreds of loci from the nuclear genome.

Hybridization‐based target enrichment (reviewed in Lemmon and Lemmon, 2013; McKain et al., 2018) is emerging as a useful reduced‐representation method for plant phylogenomics, in part because it can be used to retrieve hundreds of low‐ to single‐copy nuclear (LSCN) genes for resolving both deep and shallow relationships that may otherwise remain recalcitrant to fewer or more conserved markers (e.g., Villaverde et al., 2018). The method is also suitable for lower‐quality template DNAs, such as those extracted from herbarium specimens (Lemmon and Lemmon, 2013; Hart et al., 2016; Léveillé‐Bourret et al., 2018; McKain et al., 2018; Vatanparast et al., 2018; Villaverde et al., 2018). Such low‐quality templates may exhibit reduced sequencing success using PCR and Sanger sequencing (e.g., Chau et al., 2018). This is particularly important for groups such as *Dioscorea* that are large and geographically widespread (Wilkin et al., 2005), with few species in wide cultivation. Genomic targets of hybridization‐based enrichment approaches can be recovered using “universal” baits optimized across broad taxon sets (e.g., the Angiosperm v.1 kit of Buddenhagen et al., 2016; the Angiosperms‐353 probe set of Johnson et al., 2018; the fern bait kit of Wolf et al., 2018) and using “taxon‐specific” baits designed for a focus group, ranging in taxonomic scale from populations (e.g., *Euphorbia balsamifera* Aiton; Villaverde et al., 2018) to a variety of higher‐level taxa (e.g., *Inga* Mill., Nicholls et al., 2015; Asclepiadoideae, Weitemier et al., 2014; Asteraceae, Mandel et al., 2014). The increasing availability of public transcriptome data and pipelines for LSCN gene discovery and bait development that require relatively few genomic resources can enable clade‐specific bait design, making phylogenomic research in groups of interest more attainable (see McKain et al., 2018 for details).

Large samples of nuclear genes from different linkage groups retrieved by target enrichment can be used for species‐tree inference, by assuming that any discordances among individual gene trees are due to incomplete lineage sorting (e.g., Mirarab et al., 2014). This may be particularly useful in rapid evolutionary radiations (Nicholls et al., 2015), and especially relevant for elucidating relationships in recent lineages such as Enantiophyllum, the largest and most economically important major clade of *Dioscorea*; this clade has ~80 species (Prain and Burkill, 1938) and a median estimated crown age of 19.4 mya (Viruel et al., 2016). Various methods are suitable for phylogenomic inference. For example, individual gene trees can be reconciled by using approaches that account for coalescence differences to infer a species tree (e.g., Wickett et al., 2014; Nicholls et al., 2015). Major inconsistencies among gene trees may also occur due to hybridization/introgression and incorrect orthology vs. paralogy assignment (e.g., Maddison, 1997). When associated gene‐flow levels are low, the former problem may be accommodated by methods that were designed to deal with incomplete lineage sorting (e.g., ASTRAL; Solís‐Lemus et al., 2016). Target‐enrichment methods may simplify orthology assignment when they focus on retrieving LSCN genes that tend to be repeatedly restored to single‐copy status (diploidization) after small‐ and large‐scale duplication events. Although orthology of target loci is not guaranteed for different taxa, gene‐tree comparisons or sequence similarity methods may additionally be used for cross‐verification of orthology (e.g., Johnson et al., 2016). Here we used the relatively limited genomic resources currently available for *Dioscorea* and Dioscoreales, supplemented with new transcriptome data, to design custom baits for retrieving hundreds of LSCN genes. We applied this new *Dioscorea*‐specific target enrichment panel to a pilot taxon set that is stratified to be representative of all major clades in the genus, and that includes some of the major crops and potential CWRs, in order to evaluate its gene recovery performance on both ingroup and outgroup Dioscoreales. We used our pilot taxon sampling to test the suitability of our enrichment panel for recovering different levels of relationship, including: (1) predicting currently elusive relationships among most major *Dioscorea* clades, and (2) evaluating previously proposed hypotheses about wild relatives of a major yam crop, *D. alata*.

## METHODS

### Bait design strategy to retrieve low‐ to single‐copy nuclear genes from *Dioscorea* and relatives

We identified putative LSCN genes as candidates for bait design using four Dioscoreales transcriptomes and two monocot reference genomes. We first obtained assembled transcriptomes of *Dioscorea* sp. (Sequence Read Archive [SRA] accession ERR364396, Wickett et al., 2014; note that this taxon is published as *D. villosa* L. but is likely to have been misidentified, see below) and *Tacca chantrieri* André (J. Leebens‐Mack, University of Georgia, unpublished data), and used Trinity v. 2.4.0 (Grabherr et al., 2011; Haas et al., 2013) with default settings to generate de novo transcriptome assemblies for *D. alata* (SRX652481; Wu et al., 2015) and *D. composita* Hemsl. (SRR1525775; Wang et al., 2015). We ran two separate analyses using MarkerMiner 1.0 (Chamala et al., 2015) with these four unfiltered transcriptome assemblies as input sequences, using either *Oryza sativa* L. (Poales; a default reference in MarkerMiner) or *Xerophyta viscosa* Baker (Pandanales; as a manually added reference, Costa et al., 2017) to identify LSCN genes. Briefly, MarkerMiner identifies putative LSCN genes by first employing independent reciprocal BLAST (Altschul et al., 1990, 1997) analyses between input transcripts and a user‐specified reference proteome (*Oryza* L. and *Xerophyta* Juss. in our case) to obtain putative ortholog pairs. MarkerMiner then performs reciprocal BLAST analyses between the latter putative ortholog pairs and a set of user‐specified LSCN gene references (*Oryza* and *Xerophyta* in our case) as a final filter to only retain putative ortholog pairs with putative low‐ to single‐copy status. MarkerMiner only reports the top scoring transcripts from the latter BLAST analyses as putatively orthologous LSCN genes; if additional hits are obtained, these are reported as “secondary transcripts” that may represent splice isoforms, putative paralogs, or partially assembled transcripts, all of which we did not consider for bait design (see Chamala et al., 2015 for a complete algorithm). We added *Xerophyta* as a reference taxon in MarkerMiner, using procedures outlined in Johnson (2017a) to create relevant reference files, one containing nucleotide sequences for all genes with introns masked as Ns, and another containing their translation into amino acids. We then used the amino acid–based file to create a BLAST database using the makeblastdb application. We identified putative LSCN genes from the *Xerophyta* transcriptome (Costa et al., 2017) by using it as the input, with *Oryza* as the proteome and LSCN gene reference; MarkerMiner identified 555 putatively LSCN genes for the former taxon. For bait development, we selected a subset of 260 genes for which MarkerMiner produced top reciprocal BLAST hits to the three *Dioscorea* transcriptomes, and to the *Tacca* J. R. Forst. & G. Forst. transcriptome when applicable. We then purchased custom baits designed and produced by Arbor Biosciences (Ann Arbor, Michigan, USA), based on selected LSCN genes, in the form of a myBaits enrichment kit comprising 20,000, 100‐bp in‐solution biotinylated baits with 3× tiling density. We submitted 1288 target coding sequences from 260 genes with a total length of 2,459,581 bp for bait panel manufacture; these constituted three or four different versions of the target loci, obtained from MarkerMiner analyses (bait sequences are available in the Dryad Digital Repository [https://doi.org/10.5061/dryad.4n2fk97]; Soto Gomez et al., 2019). The final bait panel consists of 17,120 baits for targeting 260 LSCN genes with lengths between 947–3440 bp (mean 1699 bp, excluding introns) in the *Oryza* and *Xerophyta* references, and the total length of targeted coding sequence is  441,717 bp. A summary of individual gene annotations and lengths is provided in the target gene characterization file in the Dryad Digital Repository (Soto Gomez et al., 2019). We checked for target gene overlap between the *Dioscorea*‐specific panel designed here and the universal kits of Buddenhagen et al. (2016; Angiosperm v.1 kit) and Johnson et al. (2018; Angiosperm‐353 probe set) using Geneious R11 (www.geneious.com) and BLAST (Altschul et al., 1990, 1997). We used the map‐to‐reference function in Geneious with sensitivity set to highest/slow and otherwise using default settings, and the BLAST application blastn with default settings; in both programs we used target gene sequences used for bait design for each of the universal kits as references and those from our kit as queries.

### Taxon sampling for phylogenetic analysis

We selected 25 Dioscoreales taxa to test the baits, focusing on *Dioscorea*. This pilot sample set consists of 24 species of *Dioscorea* and *Trichopus zeylanicus* Gaertn. (Appendix S1). In order to assess the retrieved genes in the broader context of *Dioscorea* phylogeny, we added corresponding sequences from four *Dioscorea* leaf transcriptomes that we also generated (*D. cochleariapiculata* De Wild., *D. communis* (L.) Caddick & Wilkin, *D. soso* Jum. & H. Perrier, *D. sylvatica* Eckl.; Appendix S1; see below for methodology), five additional published *Dioscorea* transcriptomes (*D. alata*, SRR3938623; *D. nipponica* Makino, SRR5457049; *D. cayenensis*, SRR3938611; *D. trifida* L. f., SRR3938635; *D. zingiberensis* C. H. Wright, SRR1218151), and the four transcriptomes used for bait design (i.e., *D. alata*,* D. composita*,* Dioscorea* sp., and *Tacca chantrieri*). The resulting 38‐taxon matrix represents all major clades of *Dioscorea* (see Viruel et al., 2016, 2018) and two outgroup taxa (*Tacca chantrieri* and *Trichopus zeylanicus*).

### Library preparation and target enrichment

We prepared whole‐genome shotgun sequencing libraries for the 25 taxa used for target enrichment by using half‐volume reactions of the NEBNext Ultra II DNA Library Prep Kit for Illumina (New England Biolabs, Ipswich, Massachusetts, USA). We used 200 ng of starting DNA and sheared it to 350‐bp fragments with a Covaris ME220 sonicator (Covaris Inc., Woburn, Massachusetts, USA) for library preparation. We size‐selected libraries to obtain 300–400‐bp fragments, which were then amplified with six PCR cycles using the NEBNext Dual Index Primers Set I (New England Biolabs) as amplification primers. We quantified libraries using Qubit (Thermo Fisher Scientific Inc., Waltham, Massachusetts, USA) and verified fragment sizes using a TapeStation (Agilent Technologies, Santa Clara, California, USA). We enriched the 25 genomic libraries for target genes by grouping them with equal concentrations in four distinct pools (six or seven samples at equal concentrations per 1000‐ng pool). We pooled samples based on phylogenetic proximity (i.e., more closely related taxa) and DNA concentrations of individual libraries, which ranged from 11 to 80 ng/μL (Table 1). We used half‐volume reactions of the target capture kit for each pool, and otherwise followed the manufacturer protocol to enrich each pool at 65°C for 16–20 h. We then multiplexed two pools per lane for sequencing on an Illumina MiSeq platform using the v2 Micro Kit to obtain 150‐bp paired‐end reads.

**Table 1:** Capture efficiency for the 25 taxa enriched using *Dioscorea*‐specific baits, including DNA concentration of starting library (ng/μL) and designated enrichment pool.

Species	Starting library concentration (Pool no.)	Quality‐filtered paired reads^a^	Enrichment efficiency (% reads on target)	No. of assembled target genes (%)^b^	Recovered % of full target gene set length^c^	Gene tree occupancy (%)^d^	Total no. of bp retrieved by HybPiper	No. of bp included in analyses
*Dioscorea antaly*	78.6 (P4)	576,773	57.4	256 (98.5)	91.5	257 (97.3)	404,256	308,524
*Dioscorea birmanica*	40.6 (P3)	841,789	53.7	258 (99.2)	93.5	260 (98.5)	413,007	310,035
*Dioscorea calcicola*	51 (P4)	2,622,322	56.3	259 (99.6)	96.6	257 (97.3)	426,513	305,152
*Dioscorea campestris*	32.2 (P2)	1,125,117	16.8	257 (98.8)	83.8	259 (98.1)	369,975	301,896
*Dioscorea caucasica*	46.2 (P3)	2,829,147	47.1	253 (97.3)	81	250 (94.7)	357,693	288,868
*Dioscorea communis* 2	42.8 (P3)	791,550	56.8	258 (99.2)	92.1	258 (97.7)	406,539	307,100
*Dioscorea cordata*	57 (P4)	469,374	42.1	256 (98.5)	88.5	257 (97.3)	390,870	307,249
*Dioscorea daunea*	29.8 (P2)	893,672	12.5	252 (96.9)	78.9	252 (95.5)	348,579	292,679
*Dioscorea glandulosa*	11.1 (P1)	875,971	15.2	257 (98.8)	83.9	260 (98.5)	370,539	301,947
*Dioscorea hombuka*	16.6 (P1)	787,302	19.5	253 (97.3)	83.5	254 (96.2)	368,631	299,109
*Dioscorea kituiensis*	24.2 (P2)	990,417	8.9	252 (96.9)	68.6	246 (93.2)	302,829	253,766
*Dioscorea mayottensis*	37 (P2)	966,336	19.4	258 (99.2)	88.4	261 (98.9)	390,342	310,042
*Dioscorea membranacea*	58.4 (P4)	721,396	35.1	255 (98.1)	80.9	252 (95.5)	357,378	295,031
*Dioscorea minima*	16.1 (P1)	505,670	12.3	157 (60.4)	38	151 (57.2)	167,802	143,767
*Dioscorea* ×*monandra*	24.2 (P2)	823,029	11.9	245 (94.2)	71.4	245 (92.8)	315,117	274,679
*Dioscorea nitens*	13.6 (P1)	824,231	13.5	249 (95.8)	76.3	251 (95.1)	337,113	287,438
*Dioscorea nummularia*	77.2 (P4)	338,083	56.2	259 (99.6)	92.2	260 (98.5)	407,328	311,701
*Dioscorea orthogoneura*	23.2 (P1)	1,056,815	11.9	257 (98.8)	88.5	256 (97)	390,999	288,009
*Dioscorea pentaphylla*	80.4 (P4)	341,113	47.3	257 (98.8)	85.6	258 (97.7)	377,931	305,668
*Dioscorea pohlii*	74.6 (P3)	258,300	60.5	259 (99.6)	94.8	259 (98.1)	418,878	297,467
*Dioscorea prazeri*	29 (P2)	1,489,369	5.4	244 (93.8)	61.7	241 (91.3)	272,439	245398
*Dioscorea rockii*	24 (P1)	783,195	6.5	198 (76.2)	45.5	195 (73.9)	201,048	183,614
*Dioscorea sansibarensis*	39.4 (P3)	1,000,845	60.7	259 (99.6)	93.3	260 (98.5)	412,221	311,542
*Dioscorea tentaculigera*	11.8 (P1)	686,737	16.7	257 (98.8)	81.4	255 (96.6)	359,280	295,297
*Trichopus zeylanicus*	52.2 (P3)	389,519	45.4	151 (58.1)	24.2	149 (56.4)	107,082	97,020

a Number of reads produced after quality trimming using Trimmomatic (see text for details).

b Percentage calculated based on the 260 low‐ to single‐copy nuclear genes targeted using baits.

c Percentage calculated from the total number of on‐target nucleotides retrieved per sample, relative to the full length of the reference target gene set.

d Percentage calculated based on the 264 low‐ to single‐copy nuclear genes in the final data set.

### Transcriptome sequencing

We obtained leaf transcriptomes from four *Dioscorea* species by collecting three young leaves from one individual per taxon, freezing them in liquid nitrogen within 15 min of harvesting. We shipped tissue on dry ice to Novogene Corporation Inc. (Sacramento, California, USA) for RNA extraction using the RNAprep Pure Plant Kit (Polysaccharides & Polyphenolics‐rich; TIANGEN Biotech, Beijing, China), library preparation using the NEBNext Ultra RNA Library Prep Kit for Illumina (New England Biolabs), and sequencing. Samples were multiplex‐sequenced in one Illumina HiSeq 4000 lane including a total of eight samples to obtain 150‐bp paired‐end reads.

### Sequence processing, assembly, and alignment

We used FastQC v. 0.11.7 (Andrews, 2010) to assess the quality of Illumina raw reads from the 25 bait‐enriched samples and for nine of the 13 transcriptome‐based samples (i.e., those not used for bait design). We then used Trimmomatic v. 0.36 (Bolger et al., 2014) to remove low‐quality reads and adapter sequences. We set a minimum Phred quality score of 20 and sequence length of 110 bp for bait‐enriched samples. We applied the same Phred quality score for transcriptome samples, but used a 4‐bp sliding window and a minimum sequence length of 40 bp. We used HybPiper v. 1.2 (Johnson et al., 2016) to assemble and extract LSCN targets from all 25 bait‐enriched samples and from the nine previously unassembled transcriptomes. The reads_first.py script in HybPiper maps raw sequencing reads to the LSCN targets in a user‐provided reference set. We then used the HybPiper script retrieve_sequences.py with the dna flag, which outputs a single sequence per gene that is selected using length, similarity, and depth of coverage criteria (see below for methodology to deal with potential paralogs). Thus, including output from enrichment‐ and transcriptome‐based samples, HybPiper recovered up to 34 taxon terminals per file (the final files included sequences from the four additional transcriptomes used for bait design). We ran the HybPiper script intronerate.py on the 25 bait‐enriched samples to recover introns and intergenic sequences flanking targeted exons; we extracted both coding and non‐coding regions as a single concatenated sequence for each target gene using the retrieve_sequences.py script with the supercontig flag. We used MAFFT v. 7.305b (Katoh et al., 2002) to align individual genes using an accuracy‐oriented method that incorporated local pairwise alignment information and 1000 iterative refinement cycles with flags –localpair and –maxiterate 1000, and the –adjustdirectionaccurately flag to reverse‐complement opposite strand sequences where necessary, and used trimAl v. 1.4.1 (Capella‐Gutiérrez et al., 2009) to remove alignment columns where there were missing data for more than 20% of taxa. One gene was excluded from downstream analyses because it was too short after trimming (gene indicated in the target gene characterization file in the Dryad Digital Repository; Soto Gomez et al., 2019). We visually inspected individual gene alignments in AliView v. 1.18.1 (Larsson, 2014) to identify potentially misaligned sequences. We also generated a gene tree for each target locus using FastTree v. 2.1 (Price et al., 2010) with the general time reversible (GTR) DNA substitution model and otherwise using default settings, and used a Python script (Johnson, 2017b) to detect branch length outliers for alignment modification (outliers defined as branches longer than 25% of the total tree length for ingroup taxa and longer than 75% for outgroup taxa). We deleted regions that were difficult to align at the start and end of genes, or flanking sequence gaps, and the final alignments included coding regions only. We visually identified alignment blocks that differed between DNA‐based and transcriptome‐based sequences; these may result from post‐transcriptional modification processes, and so we deleted the divergent sections of transcriptome‐based sequences. We also obtained summary statistics on gene alignments using AMAS (Borowiec, 2016), including length, missing data, and number of parsimony informative sites (target gene summary statistics are available in the Dryad Digital Repository; Soto Gomez et al., 2019).

### Detection and filtering of potential paralogs

We detected 27 potential paralog instances; 26 of these through HybPiper paralog warnings and one by gene‐tree visual inspection (indicated in the target gene characterization file in the Dryad Digital Repository; Soto Gomez et al., 2019). In 22 of these cases, we selected one of the retrieved copies to be the most likely ortholog based on both highest percent identity to the reference gene and gene‐tree topology concordance with our current understanding of Dioscoreales phylogeny (Wilkin et al., 2005; Viruel et al., 2016, 2018). However, we interpreted five additional genes as being the result of a single early gene duplication (including one gene not identified by HybPiper as a possible paralogy instance; gene 168), and so we separated each into two paralogous genes, increasing the total number of recovered genes by five total (see Appendix S2 for complete methods for filtering potential paralogs). Therefore, the final data set consisted of 264 LSCN genes, each with an individual gene alignment and associated gene tree (available in the Dryad Digital Repository; Soto Gomez et al., 2019). We concatenated these 264 genes into a 319,788‐bp matrix using SequenceMatrix v. 100.0 (Vaidya et al., 2011). We also analyzed a partial data set that consisted of 232 LSCN genes by excluding the 32 genes with potential paralogy issues.

### Phylogenomic inference

We analyzed the recovered genes using coalescent‐based and concatenated maximum likelihood (ML) methods; the latter is likely to result in misinferences if there are instances of incomplete lineage sorting (e.g., Maddison, 1997), but we included it as a point of reference for the coalescent analysis. We used ASTRAL‐III (Zhang et al., 2018) with default settings for coalescent‐based analyses on both the complete 264‐gene data set and on a partial 232‐gene data set that excluded genes with potential paralogs, and also for estimating branch support for the resulting species trees using both local posterior probabilities (LPP; Sayyari and Mirarab, 2016) and multi‐locus bootstrapping (MLB; Seo, 2008). LPPs measure support for a quadripartition (the four clusters around each internal branch in a species tree) and are computed using the proportion of gene‐tree quartets that agree or disagree with an internal branch. For MLB on the 264‐gene (or 232‐gene) set, we first inferred 100 pseudo‐replicate trees from bootstrap analysis of individual genes using RAxML v. 8.2.11 (Stamatakis, 2014), with the GTRGAMMA DNA substitution model, in a pipeline developed by Ané (2018). ASTRAL then creates 100 replicate input data sets, each consisting of 264 (or 232) pseudo‐replicate gene trees (one tree per gene, randomly sampled without replacement) for quartet analysis. The frequency of times that branches appeared across the 100 resulting “species” trees is then summarized with a greedy consensus method to estimate MLB support values for each branch in the actual species tree; the latter tree is inferred from quartet analysis of one best “species” tree per gene.

For the concatenated ML analysis, we analyzed the 264‐gene concatenated matrix unpartitioned and partitioned by gene. We used PartitionFinder2 with the corrected Akaike Information Criterion (AICc) and the random clustering algorithm (Lanfear et al., 2016) to find the optimal DNA substitution model for the unpartitioned analysis, and to identify and combine sets of genes that did not have significantly different models for the partitioned analysis. PartitionFinder2 found GTR+I+G as the optimal model for unpartitioned analyses; for partitioned analyses the program retained 115 partitions, derived from 264 initial gene partitions, selecting GTR+I+G or GTR+G as the best substitution model in all cases, and we used the final data partitioning scheme for phylogenetic inference (see the gene partitioning file in the Dryad Digital Repository; Soto Gomez et al., 2019). We did not attempt more complex methods, such as ML analyses that include codon partitions or that use amino acid substitution models, as the sequences are all closely related (unpartitioned and partitioned ML analyses produced fully consistent results, and so we focused presentation of the results on the latter). We conducted ML analyses in RAxML using 20 independent searches for the best tree and estimated branch support using 500 bootstrap replicates, applying the GTR+G approximation for all data partitions, as the gamma parameter (*G*) may adequately accommodate the invariant sites parameter (*I*) (Yang, 2006). We considered strongly supported branches to have at least 0.95 LPP and 90% bootstrap support, and poorly supported ones to have less than 0.90 LPP and 70% bootstrap support.

## RESULTS

### Gene retrieval using target enrichment methods vs. transcriptome‐based data

**Figure 1:**
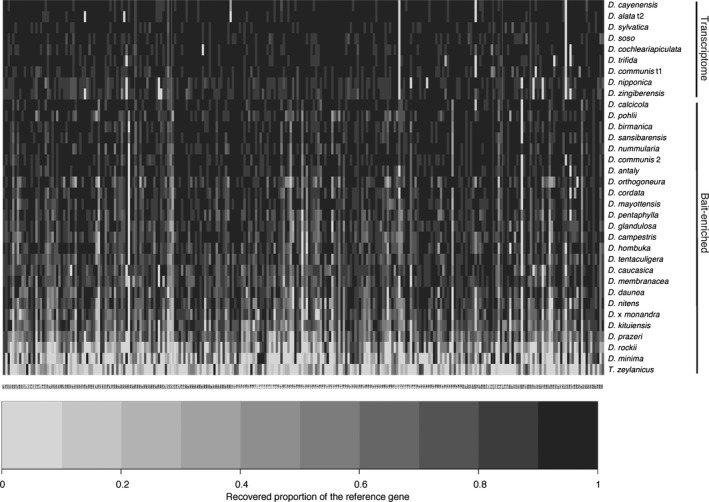
Proportion of total reference gene length recovered per gene and taxon. Rows correspond to taxa, and columns correspond to a gene target. Target enrichment using the *Dioscorea*‐specific baits designed here was performed on 25 taxa. Gene recovery for nine species with transcriptome‐based data (not including the four transcriptomes used for bait design) is also shown here, see upper set of taxa in figure. Recovered proportions of the reference gene are indicated by shading.

On average, we obtained 922,847 raw reads per enriched sample, with values ranging from 258,300 (*D. pohlii* Griseb.) to 2,830,650 (*D. caucasica* Lipsky). Over 96.5% of recovered reads were retained for all enriched samples after removing low‐quality bases and adapter sequences. We successfully recovered >243 (93.8%) of the 260 target genes from 22 of the 25 bait‐enriched samples (note that gene recovery percentages here and below are based on the 260 genes for which baits were designed, including the gene that was later excluded due to its short length after quality trimming, and exclude the five additional genes obtained through paralog analysis; Table 1). Only three samples (outgroup taxon *Trichopus zeylanicus*; ingroup taxa *D. minima* B. L. Rob. & Seaton and *D. rockii* Prain & Burkill) had lower gene recoveries, with 151 (58.1%), 157 (60.4%), and 198 (76.2%) genes, respectively (Table 1). Average enrichment efficiency, measured as the proportion of reads that HybPiper mapped to gene targets, was 31.6% (5.4–60.7%), and was above average (45.4%) for outgroup taxon *T. zeylanicus*. We recovered >80% of the full length of the reference target gene set from 17 of the 25 enriched taxa (24.2–96.6%, average of 78.6%), and only three samples had <50% recovery (outgroup taxon *T. zeylanicus*, 24.2%; ingroup taxa *D. minima*, 38% and *D. rockii*, 45.5%). The recovered proportion of the full reference gene length varied by gene and by taxon, but we retrieved ≥75% of the sequence length for most genes (153–244 genes) and taxa (20 of 25 taxa; Fig. 1). Outgroup taxon *T. zeylanicus* and ingroup taxa *D. minima*,* D. rockii*, and *D. prazeri* Prain & Burkill were the only taxa for which recovery of ≥75% of the sequence length was possible for fewer than 100 genes (14, 53, 57, 87, respectively; Fig. 1). There were 86 genes for which we recovered sequences from all 25 bait‐enriched taxa and 90 genes for which we recovered sequences from 24 taxa; there were only five genes for which the baits did not recover any sequences from more than 10 taxa (again, considering only the 260 genes for which baits were designed). After alignment quality trimming, we included an average of 276,920 bp (median 297,467 bp) per bait‐enriched taxon in phylogenetic analyses (Table 1), which represents 62.7% of the full length (441,626 bp) of the reference target gene set. We recovered an average of 1390 bp of exon‐flanking intron and intergenic sequences per locus (29–5108 bp, SD = 816 bp) from the 25 bait‐enriched taxa, after alignment and quality trimming, excluding gaps (note that our analyses focus on coding regions; lengths of retrieved coding and non‐coding sequences are available in the Dryad Digital Repository; Soto Gomez et al., 2019).

We obtained more than 250 (96.5%) of the 260 target genes from the nine taxa represented by previously unassembled transcriptomes (i.e., not including the four transcriptomes used for bait design; Table 2). We recovered ≥75% of the full target length for most genes (242–255 genes) in all nine transcriptomes (Table 2). After alignment quality trimming, we included an average of 305,952 bp (median 307,352 bp) per transcriptome‐based taxon in phylogenetic analyses, which represents 69.3% of the full length of the reference target gene set. Overall, individual gene alignments were on average 1204 bp long (144–3169 bp) and had 5.2% missing data (0–15.7%). The proportion of parsimony informative sites ranged from 14% to 38% (24% average; target gene summary statistics are available in the Dryad Digital Repository; Soto Gomez et al., 2019). The range of gene tree occupancy was 14–38 taxa, but most (213) gene trees included at least 35 of the 38 sampled taxa (Tables 1, 2; target gene summary statistics file in the Dryad Digital Repository; Soto Gomez et al., 2019).

**Table 2:** Target gene retrieval for nine taxa using transcriptome‐based data (excluding four transcriptomes used for bait design).

Species^a^	Quality‐filtered paired reads^b^	Enrichment efficiency (% reads on target)	No. of assembled target genes (%)^c^	Recovered % of full target gene set length^d^	Gene tree occupancy (%)^e^	Total no. of bp retrieved by HybPiper	No. of bp included in analyses
*Dioscorea alata* t2	35,322,722	1.8	254 (97.7)	95.3	254 (96.2)	420,909	306,180
*Dioscorea cayenensis*	59,713,857	1.8	257 (98.8)	95.5	257 (97.3)	421,914	308,395
*Dioscorea cochleariapiculata*	74,192,635	2.1	256 (98.5)	92.9	257 (97.3)	410,295	308,374
*Dioscorea communis* t1	61,649,891	2.2	257 (98.8)	91.9	258 (97.7)	405,759	307,352
*Dioscorea nipponica*	20,582,868	1.7	252 (96.9)	87.5	252 (95.5)	386,604	300,421
*Dioscorea soso*	60,869,766	2.3	258 (99.2)	94.3	258 (97.7)	416,262	309,712
*Dioscorea sylvatica*	56,326,258	2.3	258 (99.2)	95.1	258 (97.7)	420,039	309,733
*Dioscorea trifida*	32,346,683	1.7	254 (97.7)	92.5	255 (96.6)	408,300	305,784
*Dioscorea zingiberensis*	61,696,853	1.3	251 (96.5)	87.2	250 (94.7)	385,308	297,614

a The “t” label for *D. alata* and *D. communis* indicates they are transcriptome‐based sequences; both taxa are represented by an additional individual in phylogenomic analyses here, *D. alata* t1 (used for bait design and therefore not included in gene recovery analyses), and *D. communis* 2 (a DNA‐based, target‐enriched sample, see Table 1).

b Number of reads produced after quality trimming using Trimmomatic (see text for details).

c Percentage calculated based on the 260 low‐ to single‐copy nuclear genes targeted using baits.

d Percentage calculated from the total number of on‐target nucleotides retrieved per sample, relative to the full length of the reference target gene set.

e Percentage calculated based on the 264 low‐ to single‐copy nuclear genes in the final data set.

### Target gene overlap and retrieval using *Dioscorea*‐specific vs. universal baits

Our kit recovered 99.6% (259) of target genes for *D. calcicola* Prain & Burkill (Table 1) and >50% of the target length for 98.8% (257) of these (Fig. 1). *Dioscorea calcicola* is the sole member of Dioscoreales that was also examined by Johnson et al. (2018) using their Angiosperm‐353 kit, which in comparison recovered sequences from 76% (269) of the target genes for this species and >50% of the target length for 43% (152) of these genes (Johnson et al., 2018). No Dioscoreales taxa were enriched using the Angiosperm v.1 kit (Buddenhagen et al., 2016), precluding comparison. Of the 260 loci targeted by our *Dioscorea*‐specific kit, 23 genes overlap with the Angiosperm‐353 probe set (Johnson et al., 2018) and 21 with the Angiosperm v.1 kit (Buddenhagen et al., 2016); six loci overlap across all three kits (see the two comparison files in the Dryad Digital Repository for lists of overlapping genes among the three kits; Soto Gomez et al., 2019). Our kit on average recovered 95% (70–100%) of the target sequence length from *D. calcicola* for the 23 genes that overlap with the Angiosperm‐353 probe set; it retrieved most (16) genes in full, 90–93% the length of four genes and 70–86% the length of three genes. The Angiosperm‐353 probe set on average recovered 37% (0–90%) of the length of overlapping target genes from *D. calcicola* and failed for three genes (information for individual genes is available in the Dryad Digital Repository; Soto Gomez et al., 2019).

### Phylogenetic relationships inferred for the pilot taxon set of *Dioscorea* species

**Figure 2:**
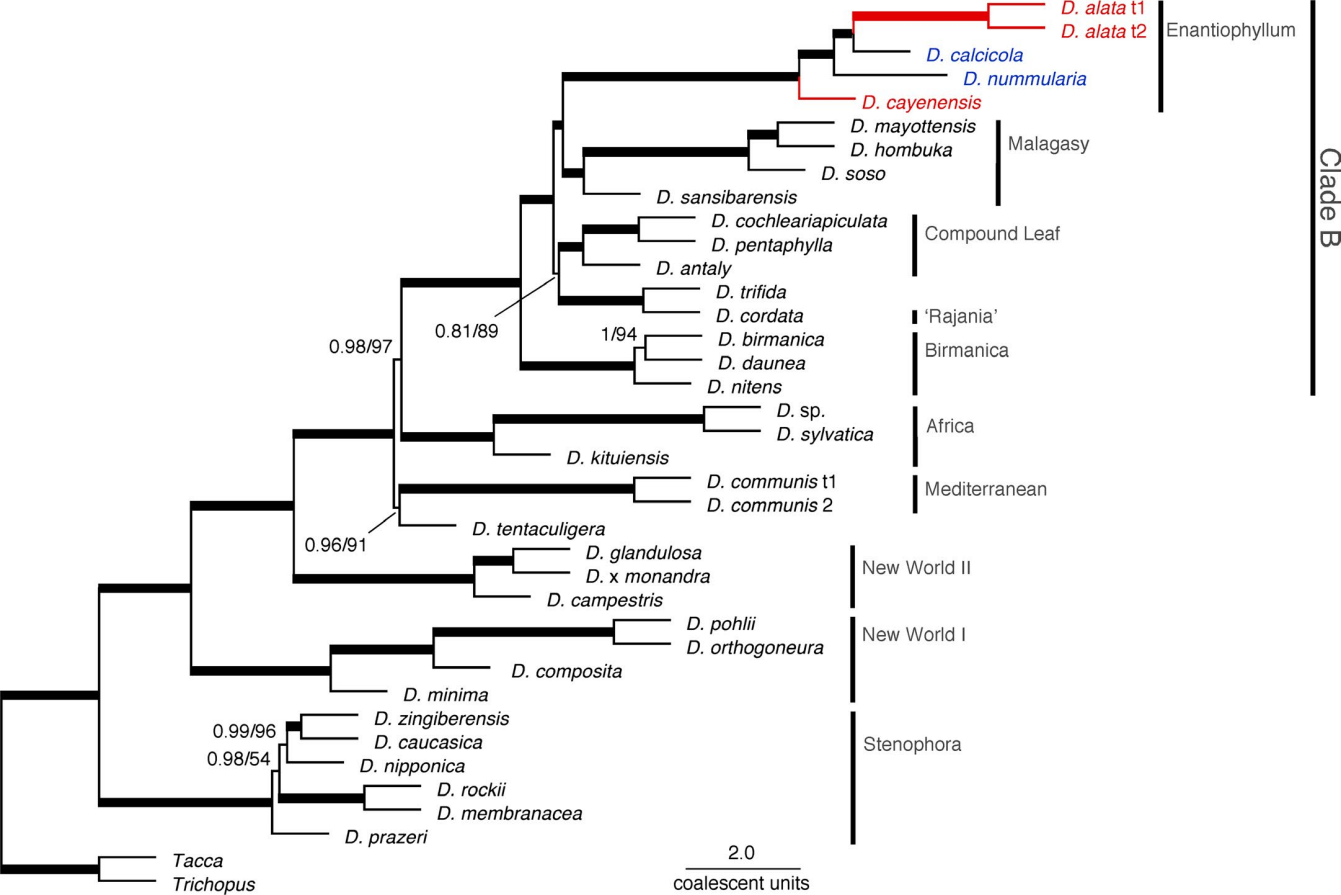
Phylogenetic relationships in *Dioscorea* inferred from coalescent‐based analyses of 264 genes recovered using target enrichment with the *Dioscorea*‐specific baits designed here. Values next to branches are local posterior probabilities (LPP) and multilocus bootstrap support (MLB), respectively; thick branches have 1.0 LPP and 100% bootstrap support. Lineages in red are major crops; blue‐labeled taxa (*D. calcicola*,* D. nummularia*) are previously identified crop wild relatives of the yam crops sampled here. Scale bar shows coalescent units for internal branches (not estimated by ASTRAL for terminal branches).

Most inferred relationships for our pilot taxon sampling for *Dioscorea* were strongly supported and consistent across both coalescent‐based (Fig. 2) and concatenated ML (Appendices [Link], [Link]) analyses, with highly consistent LPP and MLB support for the former analysis. The quartet score for the coalescent‐based tree, which is the proportion of gene‐tree quartets also present in the species tree, was 87.5%. The monophyly of major *Dioscorea* clades, and the relationships among them, were consistent and most were strongly supported across analyses (Fig. 2, Appendices [Link], [Link]). Only the major clade comprising the so‐called “compound leaf” clade and its sister group *D. trifida–D. cordata* (L.) Raz (see Fig. 2 for clade names and composition) was not strongly resolved: the entire clade was recovered across the two inference methods, but had low (0.81) support from ASTRAL LPP estimates (Fig. 2), and moderate support from ASTRAL MLB (89%; Fig. 2) and partitioned ML (88%; Appendix S3) analyses.

We also resolved most species‐level relationships with strong support in coalescent‐based and ML analyses. The placement of the two major yam crops, *D. alata* and *D. cayenensis*, was strongly resolved within the Enantiophyllum clade: *D. alata* formed a clade with *D. calcicola*, its previously proposed secondary gene pool CWR, and their sister group in turn was *D. nummularia* Lam., a previously proposed primary gene pool CWR of *D. alata* (Vincent et al., 2013; see below). *Dioscorea* sp., published as *D. villosa* in Wickett et al. (2014), is the strongly supported sister group of *D. sylvatica* in the “Africa clade,” with nearly identical branch lengths in ML analyses (Appendices [Link], [Link]). However, *D. villosa* is actually part of the Stenophora clade (Viruel et al., 2016); the latter placement is supported in unpublished analyses of a taxonomically verified sample of this species using the bait set developed here (J. Viruel, Royal Botanic Gardens, Kew, unpublished data). One of us (P.W.) assessed an image of the vouchered specimen (132728, ALTA; University of Alberta Vascular Plant Herbarium) for the *Dioscorea* sample (project sample code OCWZ) used in Wickett et al. (2014) to re‐assess its identification. Despite the limited number of diagnostic characters available from the image, a *D. sylvatica* identification is convincing, and so transcriptome data for this taxon likely originated from a misidentified individual of that species. Interspecific relationships remained uncertain only in the Stenophora clade; *D. rockii* and *D. membranacea* Pierre ex Prain & Burkill formed a strongly supported sister group, but multiple species in this clade had inconsistent relationships between the coalescent and concatenated analyses, suggesting episodes of incomplete lineage sorting for several closely related taxa in the clade.

## DISCUSSION

Hybridization capture–based technologies are enabling the retrieval of hundreds of nuclear markers from diverse plant species for resolving previously intractable relationships (e.g., Sass et al., 2016; Wolf et al., 2018). Target enrichment using universal baits can have high enrichment success across broad taxonomic scales. For example, Buddenhagen et al. (2016) recovered >90% of targets from a 53‐taxon angiosperm‐wide sampling using their universal Angiosperm v.1 kit; Johnson et al. (2018) recovered on average 80% of targets from a 42‐taxon angiosperm‐wide sampling and found that target recovery was not influenced by the degree of relatedness between the enriched sample and taxa used to design their universal Angiosperm‐353 probe set. However, taxon‐specific kits may recover a higher proportion of gene targets and overall target length, due to high complementarity between baits and focus taxa (Folk et al., 2015). This is consistent with our findings, as we recovered sequences from 99.6% of gene targets for *D. calcicola* (Table 1) here, and the Angiosperm‐353 probe set recovered 76% for this taxon (Johnson et al., 2018). Furthermore, for a set of 23 genes in common to our study, the average recovery of the target length for *D. calcicola* was 95% (70–100%) using our *Dioscorea‐*specific baits and 37% (0–90%) using the Angiosperm‐353 probe set (information for individual genes is available in the Dryad Digital Repository; Soto Gomez et al., 2019). In future, it would be useful to sample additional taxa using their and our kits for more refined comparisons.

Efficient enrichment using universal markers requires that target loci are moderately conserved across the target group (Buddenhagen et al., 2016) and so may tend to be shorter and less variable compared to custom markers (Kadleck et al., 2017; Chau et al., 2018), if only target sequences (exons) are considered. In our study, the average target length of the *Dioscorea‐*specific kit was 1699 bp. This is comparable to 1362 bp for a *Heuchera* L. kit (Folk et al., 2015), 1667 bp for a Leguminosae kit (Vatanparast et al., 2018), and ~1645 bp for a *Euphorbia* L. kit (Villaverde et al., 2018). However, average target length was shorter (820 bp) for *Buddleja* L.‐specific locus sets compared to three different non‐taxon‐specific locus sets (1267 bp; Chau et al., 2018). In contrast, the average target length for the universal Angiosperm‐353 and Angiosperm v.1 kits was 738 bp and 343 bp, respectively.

Average exon site variability (calculated from the number of sites in an alignment that show variation, including those that are parsimony informative and uninformative) of 42% from our *Dioscorea*‐specific kit (target gene summary statistics are available in the Dryad Digital Repository; Soto Gomez et al., 2019) was higher than the average 14.6% variability reported for the Angisoperm‐353 probe set for four selected genera (*Linum* L., *Neurachne* R. Br., *Oenothera* L., and *Portulaca* L.; Johnson et al., 2018), 18.5% from the Angiosperm v.1 kit for Cyperaceae (Léveillé‐Bourret et al., 2018), and 30.6% from three different non‐taxon‐specific locus sets for *Buddleja* (Chau et al., 2018). However, target enrichment methods can also consider the so‐called “splash zone” capture in regions flanking target exons, that consist of intronic and intergenic sequence (Weitemier et al., 2014; Johnson et al., 2016, 2018). These non‐coding regions are expected to be much less constrained by purifying selection and so may be useful for shallower phylogenetic applications (Buddenhagen et al., 2016), including at the population level (Johnson et al., 2016, 2018), although alignment (homology assessment) may also be more difficult than with exon regions. Comparisons of the relative utility and ease of use of flanking regions from universal kits compared to the sort of taxon‐specific exon targeting described here (we focused exclusively on coding regions) would be useful in future. A further potential benefit of custom bait design based on closer relatives of the target group is that it may help avoid targeting genes with lineage‐specific duplications (Kadlec et al., 2017), while still being applicable across a fairly broad range of related taxa (Kadlec et al., 2017; Villaverde et al., 2018).

### Capture efficiency across phylogenetic scale in *Dioscorea*: Lessons from our pilot sampling

Using transcriptomes from Dioscoreales genera *Dioscorea* and *Tacca*, and LSCN gene references from more distantly related *Oryza* and *Xerophyta*, we produced a versatile bait kit that successfully captures most target loci from the focus group (*Dioscorea*) and one other genus in the same order (*Trichopus* Gaertn.). Our average capture efficiency (i.e., the proportion of reads that HybPiper mapped to gene target references) of 31.6% (5.4–60.7%) is comparable to or lower than that from other custom baits (26% in Zingiberales, Sass et al., 2016; 48.6% in *Euphorbia*, Villaverde et al., 2018; 54.7% in *Heuchera*, Folk et al., 2015; 59% in *Oxalis* L., Schmickl et al., 2016). Our average recovery of the total reference gene set length was relatively high at 78.6% compared to other studies using custom baits (e.g., 33.4% in Folk et al., 2015; 59.4% in Sass et al., 2016; 73% in Villaverde et al., 2018), but varied across taxa (24.2–96.6%). Baits are expected to work well up to a 30% threshold of sequence divergence to targets (Johnson et al., 2018). Therefore, variability in enrichment success in our study was likely not fully due to insufficient affinity between samples and baits (including for *Trichopus*, as its uncorrected *p*‐distance to sampled *Dioscorea* was 0–8%), but rather to differences in sample pooling strategy, starting DNA quality and quantity, library complexity, and genome size (Hart et al., 2016; Sass et al., 2016; Johnson et al., 2018). For example, we expect that lower gene recovery for *Trichopus* reported here (151 of 260 genes; Table 1) could have been improved by not pooling this outgroup taxon with *Dioscorea* taxa; baits were designed using *Dioscorea* transcriptomes and so a high proportion of them were likely sequestered by more similar ingroup samples (Villaverde et al., 2018). *Dioscorea minima* had similar gene recovery to outgroup *Trichopus* with 157 of 260 genes; the former ingroup taxon had the third lowest amount of starting DNA (1.88 ng/μL), which may have impacted enrichment success, especially if the quality of the available DNA was poor and the resulting genomic library had low complexity. Although we considered starting DNA amounts for our six to seven taxon pools, samples with low amounts and/or poor quality DNA may benefit from even smaller pool sizes or individual enrichment. Additionally, genome size data are only available for *D. alata*,* D. caucasica*,* D. cayenensis*,* D. communis*,* D. sylvatica*, and *D. zingiberensis* among sampled taxa, and so we may have pooled taxa of varying ploidy levels and genome sizes; the latter are reported to range between 0.35 and 6.75 pg in *Dioscorea* (Bennett and Leitch, 2012; Zhang et al., 2013; Šmarda et al., 2014). Johnson et al. (2018) enriched samples with larger genomes in smaller pools, and this strategy may have contributed to their finding that genome size did not have a large impact on target recovery. Sample‐pooling strategies should therefore consider amount and quality of starting DNA, phylogenetic distance to taxa upon which baits were designed (especially if baits are not universal), and ploidy level and genome size, when known.

### Custom baits resolved lower‐ and higher‐order relationships in a pilot taxon sampling

Our pilot study of *Dioscorea* was based on a relatively small but broadly representative set of taxa in order to assess how well our target enrichment panel may perform for broad samplings in the genus. We included taxa from all of the major *Dioscorea* lineages identified in previous studies (Viruel et al., 2016, 2018), as well as several groups of more closely related taxa, to assess whether we could recover different levels of relationships that remained unresolved or poorly supported in previous studies. The composition of major clades and their interrelationships mostly agreed with published findings (Wilkin et al., 2005; Hsu et al., 2013; Maurin et al., 2016; Viruel et al., 2016, 2018; Couto et al., 2018) but were consistently better supported across both coalescent‐based and ML analyses here. The tree inferred by Viruel et al. (2018) from Bayesian analyses of a matrix consisting of one nuclear gene and five plastid markers recovered lineages from the Birmanica clade as being nested within the Enantiophyllum clade with strong support (posterior probability [PP] of 1); this arrangement was moderately supported in their ML analyses. Here we confirmed the monophyly of both the Enantiophyllum and Birmanica clades with strong support across all analyses (Fig. 2, Appendices [Link], [Link]; see below for relationships to other major clades).

Our taxon sampling has the highest species overlap with earlier studies by Wilkin et al. (2005), Viruel et al. (2016, 2018), and Couto et al. (2018). There was one strongly supported conflict concerning relationships among major clades between our study and that of Viruel et al. (2018), and two additional conflicts between our study and findings from Couto et al. (2018) considering Bayesian posterior probability values in their studies (support for these three branches from ML bootstrap analyses in those studies was moderate and low, respectively). The tree inferred from combined analysis of one nuclear gene and five plastid markers by Viruel et al. (2018) reconstructed the Mediterranean clade as the sister group of clade B (0.97 PP). However, in our study, clade B is the sister group of the Africa clade, with high support across all analyses. The tree inferred from two plastid genes by Couto et al. (2018) recovered the compound leaf clade as the sister group of all other taxa in clade B (0.99 PP), but here the latter is the sister group of the Birmanica clade with strong support across all analyses. Couto et al. (2018) also placed the Enantiophyllum clade as the sister group to a clade that includes the Rajania, Birmanica, and Malagasy clades (0.96 PP), whereas in our study only the Malagasy clade is the strongly supported sister group of the Enantiophyllum clade across all analyses. Only one branch concerning major clade relationships was not consistently strongly resolved here: there was low ASTRAL LPP (0.81; Fig. 2) support, moderate ASTRAL MLB (89%; Fig. 2) support, and moderate to high ML (88–93%; Appendices [Link], [Link]) support for the clade that comprises the compound leaf clade and its sister group *D. trifida*–*D. cordata*. The length of the branch subtending the entire compound leaf plus *D. trifida*–*D. cordata* clade was relatively short in the phylogeny produced by ASTRAL, in which branch lengths represent coalescent units that directly measure discordance in gene trees. Indeed, for this latter less strongly supported branch, quartet support for the species tree topology was 39% and support for the two alternative topologies was 24% and 36%.

Gene‐tree inference uncertainty and hybridization (including subsequent introgression and homoploid or polyploid speciation) are two additional possible sources of gene‐tree and gene‐species tree discordance that our methods do not take into account. Gene‐tree estimation error is also likely to occur with most biological data sets, but may be more challenging for multi‐copy genes spanning a large number of species (Bayzid and Warnow, 2018), and so may be a more minor source of discordance in our pilot 38‐taxon LSCN gene set. Hybridization is generally not well characterized for the lineages sampled here, except for the putative hybrid *D*. ×*monandra* Hauman (*D. cienegensis* R. Knuth × *D. glomerulata* Hauman; Govaerts et al., 2018). However, natural hybridization has been documented between additional *Dioscorea* lineages (e.g., Martin and Cabanillas, 1963) and is relatively common in plants in general (Soltis and Soltis, 2009). Coalescent‐based methods such as ASTRAL may incorrectly reconstruct species trees in the presence of gene flow, as incomplete lineage sorting is not the only possible source of gene‐tree conflict (Solís‐Lemus et al., 2016). However, when gene flow levels are low, ASTRAL can estimate the true species tree with increasing accuracy as more genes are added (Solís‐Lemus et al., 2016). Interspecific gene flow can also be detected through gene‐tree discordance (Galtier and Daubin, 2008); gene flow may not be widespread in our data set, given that nearly 90% of gene‐tree quartets were also found in the species tree. Applying the *Dioscorea*‐specific kit to a more expanded sampling of the genus that includes confirmed or suspected hybrids and potential parent species will be useful for testing how effective these target loci are for detecting hybrids; this will require allele and homeolog phasing, a challenge that may be simplified by using longer‐read sequencing platforms, such as MiSeq (Wolf et al., 2018).

Resolution of species‐level relationships using nuclear markers is of particular interest in *Dioscorea* because it includes multiple major and minor crop species, and the identities of the closest CWRs of most of these remain elusive, partly due to limited taxon sampling and insufficient variability of molecular markers, most of which have been from the plastid genome (Chaïr et al., 2005, 2016; Mukherjee and Bhat, 2013; Girma et al., 2016; but see Girma et al., 2014 for SNP analysis of Guinea yam and selected CWRs) or were not sequence‐based (Terauchi et al., 1992; Malapa et al., 2005; Mignouna et al., 2005). Identifying CWRs is a significant endeavor for crop scientists (Dempewolf et al., 2014), as these taxa may represent a substantial pool of genetic diversity for traits that may be useful for future crop improvement and may be the most straightforward to cross in breeding experiments (Harlan and de Wet, 1971). We recovered *D. calcicola*, a previously proposed secondary gene pool CWR, as a strongly supported sister group of the major yam crop *D. alata* among sampled taxa, suggesting that it may be an even closer relative than its previously proposed primary gene pool CWR, *D. nummularia*. Primary gene pool CWRs are considered to be closely related (even conspecific) taxa that are fully interfertile with the crop taxon, whereas secondary gene pool CWRs represent more distantly related taxa that can be crossed to the crop taxon with more difficulty (Harlan and de Wet, 1971). These CWR groupings for *Dioscorea* were proposed in the Harlan and de Wet CWR inventory (Vincent et al., 2013) using genetic distances (Malapa et al., 2005) and phylogenetic relationships (Wilkin et al., 2005). Regardless of how CWR groupings are defined, our findings suggest that *D. calcicola* may have utility in future breeding programs for *D. alata*.

### Characterization of loci targeted by our custom *Dioscorea*‐specific enrichment panel

The MarkerMiner pipeline we used for LSCN gene selection has previously been shown to select more conserved loci compared to two other commonly used methods, at least for LSCN gene discovery in Leguminosae (Vatanparast et al., 2018). However, our data set contained sufficient parsimony informative sites (average 24%) to strongly resolve most species‐level relationships. We are currently applying our bait kit to >400 *Dioscorea* species, and preliminary phylogenomic analyses show that our approach can strongly resolve relationships using much denser sampling than that for this pilot taxon set (J. Viruel, Royal Botanic Gardens, Kew, unpublished data). Our custom nuclear gene targets may also be informative for resolving population‐level relationships, such as those elucidated by Villaverde et al. (2018) for *Euphorbia* using a custom bait kit with an average of 8% parsimony informative sites for exons and 9% for exons plus introns. In *Dioscorea*, resolution at this shallow level may be particularly useful for characterizing variability of germplasm collections of wild and cultivated yam, a goal for improved management and use of genetic resources in this crop (Mignouna et al., 2005). Using non‐coding regions (i.e., introns and intergenic regions), which we excluded here, may also be useful in this context.

The MarkerMiner pipeline we implemented here recovered fewer loci that were flagged as potential paralogy instances by HybPiper, compared to two other methods used for LSCN gene identification in Leguminosae (Vatanparast et al., 2018). We detected potential paralogy, either through HybPiper warnings or by visual inspection of all generated gene alignments and trees, in 27 (10.4%) of the 260 genes for which baits were designed; orthologs were teased apart in most cases after inspection of gene trees. When we excluded 32 potentially paralogous genes (27 potential paralogy instances plus five genes that we derived from the latter after interpreting them as cases of early duplications, see Methods, Appendix S2 and the gene characterization file in the Dryad Digital Repository; Soto Gomez et al., 2019), ASTRAL analysis of the resulting 232‐gene data set (Appendix S5) produced mostly highly supported relationships that were fully consistent with those from ASTRAL analysis of the full 264‐gene data set. This may be an underestimate of the amount paralogy, but comparisons of analyses with and without these genes confirmed that our phylogenomic inferences are robust to inclusion vs. exclusion of the potential paralogs we recovered with our kit (Fig. 2 cf. Appendix S5). Visual inspection of individual gene trees and alignments may therefore not be essential for species‐tree inference. However, investigating loci flagged by HybPiper may be useful for the potential characterization and quantification of paralogy issues. Visual inspection of gene alignments and trees may be more critical when adding transcriptome data to target enrichment data, because post‐transcriptional modifications may lead to inadvertent alignment of non‐homologous regions in some genes across these two data types. We also note that without visual inspection we may not have identified multiple instances where gene copies likely represented chimeras of orthologous and paralogous sequences.

### Conclusions

The versatile bait kit designed here recovered gene targets effectively from *Dioscorea* and one Dioscoreales outgroup taxon, enabling resolution of relationships at various taxonomic scales. Our enrichment panel will be a useful tool for phylogenomic investigations of *Dioscorea* and allies. It may be used to develop an evolutionary framework for identifying the CWRs of yam crops, or to map traits of agricultural and evolutionary interest, and potentially investigate the importance of hybridization in the evolution of the genus. It may also be more broadly applicable in phylogenomic analyses across Dioscoreales. Whether universal angiosperm kits will displace the need for custom‐designed taxon‐focused kits remains to be thoroughly assessed. However, at minimum these two approaches should be complementary for groups of interest. Our kit has minimal overlap with kits designed for broader taxonomic studies in terms of target genes; it recovers a higher fraction of genes per those examined, and the recovered portions are longer and more variable for our group of interest than a universal kit allowed for a species of *Dioscorea*. Finally, analyses based on a pilot taxon sampling predict that our panel will be suitable for phylogenomic analyses of a much denser taxonomic sampling in *Dioscorea*.

## AUTHOR CONTRIBUTIONS

M.S.G., J.V., M.B.K., S.W.G., P.W., I.J.L., F.F., and B.G. designed and retrieved the funding for the project. L.P. advised on methods and analyses. M.S.G. and J.V. conducted the study design, bait development, experimental work, and analyses. M.S.G., J.V., and S.W.G. led the writing; all coauthors collaborated on writing and revising the manuscript.

## Supporting information


**APPENDIX S1.** Specimen source information for the 25 taxa enriched using *Dioscorea‐*specific baits, and for the four newly generated transcriptomes (the latter are indicated with an asterisk).

**APPENDIX S2.** Supplementary methods for detecting and filtering potential paralogs.

**APPENDIX S3.** Phylogenetic relationships in *Dioscorea* inferred from partitioned maximum likelihood analyses on a concatenated matrix of 264 genes recovered using target enrichment with the *Dioscorea*‐specific baits designed here.

**APPENDIX S4.** Phylogenetic relationships in *Dioscorea* inferred from unpartitioned maximum likelihood analyses on a concatenated matrix of 264 genes recovered using target enrichment with the *Dioscorea*‐specific baits designed here.

**APPENDIX S5.** Phylogenetic relationships in *Dioscorea* inferred from coalescent‐based analyses of a partial 232 gene set that excluded 32 potential paralogs.

## Data Availability

Bait sequences, individual alignments for each target locus, and resulting gene trees are available on the Dryad Digital Repository (https://doi.org/10.5061/dryad.4n2fk97; Soto Gomez et al., 2019). Raw reads from sequencing of bait‐enriched samples and four newly generated transcriptomes are available in the National Center for Biotechnology Information (NCBI) Sequence Read Archive (BioProject ID PRJNA530017).

## References

[aps311254-bib-0001] Altschul, S. F. , W. Gish , W. Miller , E. W. Myers , and D. J. Lipman . 1990 Basic local alignment search tool. Journal of Molecular Biology 215: 403–410. 2231712 10.1016/S0022-2836(05)80360-2

[aps311254-bib-0002] Altschul, S. F. , T. L. Madden , A. A. Schäffer , J. Zhang , Z. Zhang , W. Miller , and D. J. Lipman . 1997 Gapped BLAST and PSI‐BLAST: A new generation of protein database search programs. Nucleic Acids Research 25: 3389–3402. 9254694 10.1093/nar/25.17.3389 PMC146917

[aps311254-bib-0003] Andrews, S. 2010 FastQC: A quality control tool for high throughput sequence data. Available at http://www.bioinformatics.babraham.ac.uk/projects/fastqc/ [accessed 12 July 2018].

[aps311254-bib-0004] Ané, C. 2018 PhyloNetwork software workshop. Website https://github.com/crsl4/PhyloNetworks.jl/wiki/TICR:-from-alignments-to-quartet-concordance-factors [accessed 10 July 2018].

[aps311254-bib-0005] Asiedu, R. , and A. Sartie . 2010 Crops that feed the world 1. Yams. Food Security 2: 305–315.

[aps311254-bib-0006] Bayzid, M. S. , and T. Warnow . 2018 Gene tree parsimony for incomplete gene trees: Addressing true biological loss. Altorithms for Molecular Biology 13: 1. 10.1186/s13015-017-0120-1 PMC5774205 29387142

[aps311254-bib-0007] Bennett, M. D. , and I. J. Leitch . 2012 Plant DNA C‐values Database–Home [online]. http://data.kew.org/cvalues/CvalServlet?querytype=2 [accessed 15 August 2018].

[aps311254-bib-0008] Bhattacharjee, R. , M. Gedil , A. Sartie , E. Otoo , D. Dumet , H. Kikuno , P. L. Kumar , and R. Asiedu . 2011 Chapter 4 *Dioscorea* *In* Kole C. [ed.], Wild crop relatives: Genomic and breeding resources. Industrial Crops, 71–96. Springer‐Verlag, Berlin, Germany.

[aps311254-bib-0009] Bolger, A. M. , M. Lohse , and B. Usadel . 2014 Trimmomatic: A flexible trimmer for Illumina sequence data. Bioinformatics 30: 2114–2120. 24695404 10.1093/bioinformatics/btu170 PMC4103590

[aps311254-bib-0010] Borowiec, M. L. 2016 AMAS: A fast tool for alignment manipulation and computing of summary statistics. PeerJ 4: e1660. 26835189 10.7717/peerj.1660 PMC4734057

[aps311254-bib-0011] Buddenhagen, C. , A. R. Lemmon , E. M. Lemmon , J. Bruhl , J. Cappa , W. L. Clement , M. Donoghue , et al. 2016 Anchored phylogenomics of angiosperms I: Assessing the robustness of phylogenetic estimates. bioRxiv 086298 [Preprint]. 28 November 2016 [cited 2 November 2018]. Available from doi: 10.1101/086298.

[aps311254-bib-0012] Capella‐Gutiérrez, S. , J. M. Silla‐Martínez , and T. Gabaldón . 2009 TrimAl: A tool for automated alignment trimming in large‐scale phylogenetic analyses. Bioinformatics 25: 1972–1973. 19505945 10.1093/bioinformatics/btp348 PMC2712344

[aps311254-bib-0013] Chaïr, H. , X. Perrier , C. Agbangla , J. L. Marchand , O. Dainou , and J. L. Noyer . 2005 Use of cpSSRs for the characterisation of yam phylogeny in Benin. Genome 48: 674–684. 16094435 10.1139/g05-018

[aps311254-bib-0014] Chaïr, H. , J. Sardos , A. Supply , P. Mournet , R. Malapa , and V. Lebot . 2016 Plastid phylogenetics of Oceania yams (*Dioscorea* spp., Dioscoreaceae) reveals natural interspecific hybridization of the greater yam (*D. alata*). Botanical Journal of the Linnean Society 180: 319–333.

[aps311254-bib-0015] Chamala, S. , N. García , G. T. Godden , V. Krishnakuma , I. E. Jordon‐Thaden , R. De Smet , and W. B. Barbazuk . 2015 MarkerMiner 1.0: A new application for phylogenetic marker development using angiosperm transcriptomes. Applications in Plant Sciences 3: 1400115. 10.3732/apps.1400115 PMC4406834 25909041

[aps311254-bib-0016] Chau, J. H. , W. A. Rahfeldt , and R. G. Olmstead . 2018 Comparison of taxon‐specific versus general locus sets for targeted sequence capture in plant phylogenomics. Applications in Plant Sciences 6: e1032. 29732262 10.1002/aps3.1032 PMC5895190

[aps311254-bib-0017] Costa, M. C. D. , M. A. S. Artur , J. Maia , E. Jonkheer , M. F. L. Derks , H. Nijveen , and B. Williams . 2017 A footprint of desiccation tolerance in the genome of *Xerophyta viscosa* . Nature Plants 3: 17038. 28346448 10.1038/nplants.2017.38

[aps311254-bib-0018] Couto, R. S. , A. C. Martins , M. Bolson , R. C. Lopes , E. C. Smidt , and J. M. A. Braga . 2018 Time calibrated tree of *Dioscorea* (Dioscoreaceae) indicates four origins of yams in the Neotropics since the Eocene. Botanical Journal of the Linnean Society 188: 144–160.

[aps311254-bib-0019] De Luca, V. , V. Salim , S. M. Atsumi , and F. Yu . 2012 Mining the biodiversity of plants: A revolution in the making. Science 336: 1658–1661. 22745417 10.1126/science.1217410

[aps311254-bib-0020] Dempewolf, H. , R. J. Eastwood , L. Guarino , C. K. Khoury , J. V. Müller , and J. Toll . 2014 Adapting agriculture to climate change: A global initiative to collect, conserve, and use crop wild relatives. Agroecology and Sustainable Food Systems 38: 369–377.

[aps311254-bib-0021] Folk, R. A. , J. R. Mandel , and J. V. Freudenstein . 2015 A protocol for targeted enrichment of intron‐containing sequence markers for recent radiations: A phylogenomic example from *Heuchera* (Saxifragaceae). Applications in Plant Sciences 3: 1500039. 10.3732/apps.1500039 PMC4542943 26312196

[aps311254-bib-0022] Galtier, N. , and V. Daubin . 2008 Dealing with incongruence in phylogenomic analyses. Philosophical Transactions of the Royal Society B: Biological Sciences 363: 4023–4029. 10.1098/rstb.2008.0144 PMC2607408 18852109

[aps311254-bib-0023] Girma, G. , K. E. Hyma , R. Asiedu , S. E. Mitchell , M. Gedil , and C. Spillane . 2014 Next‐generation sequencing based genotyping, cytometry and phenotyping for understanding diversity and evolution of guinea yams. Theoretical and Applied Genetics 127: 1783–1794. 24981608 10.1007/s00122-014-2339-2

[aps311254-bib-0024] Girma, G. , C. Spillane , and M. Gedil . 2016 DNA barcoding of the main cultivated yams and selected wild species in the genus *Dioscorea* . Journal of Systematics and Evolution 54: 228–237.

[aps311254-bib-0025] Govaerts, R. , P. Wilkin , L. Raz , and O. Téllez‐Valdés . 2018 World checklist of Dioscoreaceae. Facilitated by the Royal Botanic Gardens, Kew. Website http://wcsp.science.kew.org/ [accessed 10 August 2018].

[aps311254-bib-0026] Grabherr, M. G. , B. J. Haas , M. Yassour , J. Z. Levin , D. A. Thompson , I. Amit , X. Adiconis , et al. 2011 Full‐length transcriptome assembly from RNA‐seq data without a reference genome. Nature Biotechnology 29: 644–652. 10.1038/nbt.1883 PMC3571712 21572440

[aps311254-bib-0027] Haas, B. J. , A. Papanicolaou , M. Yassour , M. Grabherr , P. D. Blood , J. Bowden , M. B. Couger , et al. 2013 De novo transcript sequence reconstruction from RNA‐seq using the Trinity platform for reference generation and analysis. Nature Protocols 8: 1494–1512. 23845962 10.1038/nprot.2013.084 PMC3875132

[aps311254-bib-0028] Harlan, J. R. , and J. M. J. de Wet . 1971 Toward a rational classification of cultivated plants. Taxon 20: 509–517.

[aps311254-bib-0029] Hart, M. L. , L. L. Forrest , J. A. Nicholls , and C. A. Kidner . 2016 Retrieval of hundreds of nuclear loci from herbarium specimens. Taxon 65: 1081–1092.

[aps311254-bib-0030] Hsu, K.‐M. , J.‐L. Tsai , M.‐Y. Chen , H.‐M. Ku , and S.‐C. Liu . 2013 Molecular phylogeny of *Dioscorea* (Dioscoreaceae) in East and Southeast Asia. Blumea 58: 21–27.

[aps311254-bib-0031] Hua, W. , W. Kong , X. Y. Cao , C. Chen , Q. Liu , X. Li , and Z. Wang . 2017 Transcriptome analysis of *Dioscorea zingiberensis* identified genes involved in diosgenin biosynthesis. Genes & Genomics 39: 509–520.

[aps311254-bib-0032] Johnson, M. G. 2017a Prepping zebrafinch genome for blackbird transcript alignments [Website https://www.evernote.com/client/web#?n=b9b6adcc-b62d-41c8-9837 ‐online]. 8d76e517e670&s=s593& [accessed 30 May 2017].

[aps311254-bib-0033] Johnson, M. G. 2017b Phyloscripts by mossmatters. Website https://github.com/mossmatters/phyloscripts/tree/master/brlenoutliers [accessed 10 March 2018].

[aps311254-bib-0034] Johnson, M. G. , E. M. Gardner , Y. Liu , R. Medina , B. Goffinet , A. J. Shaw , N. J. C. Zerega , and N. J. Wickett . 2016 HybPiper: Extracting coding sequence and introns for phylogenetics from high‐throughput sequencing reads using target enrichment. Applications in Plant Sciences 4: 1600016. 10.3732/apps.1600016 PMC4948903 27437175

[aps311254-bib-0035] Johnson, M. G. , L. Pokorny , S. Dodsworth , L. R. Botigué , R. S. Cowan , A. Devault , W. L. Eiserhardt , et al. 2018 A universal probe set for targeted sequencing of 353 nuclear genes from any flowering plant designed using k‐medoids clustering. Systematic Biology 10.1093/sysbio/syy086. PMC6568016 30535394

[aps311254-bib-0036] Kadlec, M. , D. U. Bellstedt , N. C. Le Maitre , and M. D. Pirie . 2017 Targeted NGS for species level phylogenomics: “Made to measure” or “one size fits all”? PeerJ 5: e3569. 28761782 10.7717/peerj.3569 PMC5530999

[aps311254-bib-0037] Katoh, K. , K. Misawa , K. Kuma , and T. Miyata . 2002 MAFFT: A novel method for rapid multiple sequence alignment based on fast Fourier transform. Nucleic Acids Research 30: 3059–3066. 12136088 10.1093/nar/gkf436 PMC135756

[aps311254-bib-0038] Lanfear, R. , P. B. Frandsen , A. M. Wright , T. Senfeld , and B. Calcott . 2016 PartitionFinder 2: New methods for selecting partitioned models of evolution for molecular and morphological phylogenetic analyses. Molecular Biology and Evolution 34: 772–773. 10.1093/molbev/msw260 28013191

[aps311254-bib-0039] Larsson, A. 2014 AliView: A fast and lightweight alignment viewer and editor for large data sets. Bioinformatics 30: 3276–3278. 25095880 10.1093/bioinformatics/btu531 PMC4221126

[aps311254-bib-0040] Lebot, V. 2009 Section III. Yams *In* Atherton J. and Rees A. [eds.], Tropical root and tuber crops: Cassava, sweet potato, yams and aroids, 181–275. CABI Publishing, Wallingford, United Kingdom.

[aps311254-bib-0041] Lemmon, E. M. , and A. R. Lemmon . 2013 High‐throughput genomic data in systematics and phylogenetics. Annual Review of Ecology, Evolution, and Systematics 44: 99–121.

[aps311254-bib-0042] Léveillé‐Bourret, E. , J. R. Starr , B. A. Ford , E. M. Lemmon , and A. R. Lemmon . 2018 Resolving rapid radiations within angiosperm families using anchored phylogenomics. Systematic Biology 67: 94–112. 28472459 10.1093/sysbio/syx050

[aps311254-bib-0043] Maddison, W. P. 1997 Gene trees in species trees. Systematic Biology 46: 523–536.

[aps311254-bib-0044] Malapa, R. , G. Arnau , J. L. Noyer , and V. Lebot . 2005 Genetic diversity of the greater yam (*Dioscorea alata* L.) and relatedness to *D. nummularia* Lam. and *D. transversa* Br. as revealed with AFLP markers. Genetic Resources and Crop Evolution 52: 919–929.

[aps311254-bib-0045] Mandel, J. R. , R. B. Dikow , V. A. Funk , R. R. Masalia , S. E. Staton , A. Kozik , R. W. Michelmore , et al. 2014 A target enrichment method for gathering phylogenetic information from hundreds of loci: An example from the Compositae. Applications in Plant Sciences 2: 1300085. 10.3732/apps.1300085 PMC4103609 25202605

[aps311254-bib-0046] Martin, F. W. , and E. Cabanillas . 1963 A wild hybrid of sapogenin‐bearing *Dioscorea* species. Bulletin of the Torrey Botanical Club 90: 232–237.

[aps311254-bib-0047] Martin, F. W. , and L. Degras . 1978 Tropical yams and their potential: part 6. Minor cultivated *Dioscorea* species. Agriculture Handbook Number 538. Science and Education Administration, United States Department of Agriculture in cooperation with the Agency for International Development [online]. Website https://naldc-legacy.nal.usda.gov/naldc/download.xhtml?xml:id=CAT87209435&content=PDF [accessed 20 August 2018].

[aps311254-bib-0048] Maurin, O. , A. M. Muasya , P. Catalán , E. Z. Shongwe , J. Viruel , P. Wilkin , and M. Van der Bank . 2016 Diversification into novel habitats in the Africa clade of *Dioscorea* (Dioscoreaceae): Erect habit and elephant's foot tubers. BMC Evolutionary Biology 16: 238. 27821045 10.1186/s12862-016-0812-z PMC5100304

[aps311254-bib-0049] McKain, M. R. , M. G. Johnson , S. Uribe‐Convers , D. Eaton , and Y. Yang . 2018 Practical considerations for plant phylogenomics. Applications in Plant Sciences 6: e1038. 29732268 10.1002/aps3.1038 PMC5895195

[aps311254-bib-0050] Mignouna, H. D. , M. M. Abang , and R. Asiedu . 2003 Harnessing modern biotechnology for tropical tuber crop improvement: Yam (*Dioscorea* spp.) molecular breeding. African Journal of Biotechnology 2: 478–485.

[aps311254-bib-0051] Mignouna, H. D. , M. M. Abang , N. W. Wanyera , V. A. Chikaleke , R. Asiedu , and G. Thottappilly . 2005 PCR marker‐based analysis of wild and cultivated yams (*Dioscorea* spp.) in Nigeria: Genetic relationships and implications for *ex situ* conservation. Genetic Resources and Crop Evolution 52: 755–763.

[aps311254-bib-0052] Mirarab, S. , R. Reaz , M. S. Bayzid , T. Zimmermann , M. S. Swenson , and T. Warnow . 2014 ASTRAL: Genome‐scale coalescent‐based species tree estimation. Bioinformatics 30: i541–i548. 25161245 10.1093/bioinformatics/btu462 PMC4147915

[aps311254-bib-0053] Mukherjee, P. , and K. V. Bhat . 2013 Phylogenetic relationship of wild and cultivated yam species (*Dioscorea* spp.) of India inferred from PCR–RFLP analysis of two cpDNA loci. Plant Systematics and Evolution 299: 1587–1597.

[aps311254-bib-0054] Nicholls, J. A. , R. T. Pennington , E. J. M. Koenen , C. E. Hughes , J. Hearn , L. Bunnefeld , K. G. Dexter , et al. 2015 Using targeted enrichment of nuclear genes to increase phylogenetic resolution in the neotropical rain forest genus *Inga* (Leguminosae: Mimosoideae). Frontiers in Plant Science 6: https://www.frontiersin.org/article/10.3389/fpls.2015.00710. 10.3389/fpls.2015.00710 PMC4584976 26442024

[aps311254-bib-0055] Prain, D. , and I. H. Burkill . 1938 An account of the genus *Dioscorea* in the East, Part 2: The species which tine to the right. Annals of the Royal Botanic Gardens, Calcutta 14: 211–528.

[aps311254-bib-0056] Price, M. N. , P. S. Dehal , and A. P. Arkin . 2010 FastTree 2: Approximately maximum‐likelihood trees for large alignments. PLoS ONE 5: e9490. 20224823 10.1371/journal.pone.0009490 PMC2835736

[aps311254-bib-0057] Sass, C. , W. J. D. Iles , C. F. Barrett , S. Y. Smith , and C. D. Specht . 2016 Revisiting the Zingiberales: Using multiplexed exon capture to resolve ancient and recent phylogenetic splits in a charismatic plant lineage. PeerJ 4: e1584. 26819846 10.7717/peerj.1584 PMC4727956

[aps311254-bib-0058] Sayyari, E. , and S. Mirarab . 2016 Fast coalescent‐based computation of local branch support from quartet frequencies. Molecular Biology and Evolution 33: 1654–1668. 27189547 10.1093/molbev/msw079 PMC4915361

[aps311254-bib-0059] Schmickl, R. , A. Liston , V. Zeisek , K. Oberlander , K. Weitemier , S. C. K. Straub , R. C. Cronn , et al. 2016 Phylogenetic marker development for target enrichment from transcriptome and genome skim data: The pipeline and its application in southern African *Oxalis* (Oxalidaceae). Molecular Ecology Resources 16: 1124–1135. 26577756 10.1111/1755-0998.12487

[aps311254-bib-0060] Seo, T.‐K. 2008 Calculating bootstrap probabilities of phylogeny using multilocus sequence data. Molecular Biology and Evolution 25: 960–971. 18281270 10.1093/molbev/msn043

[aps311254-bib-0061] Šmarda, P. , P. Bureš , L. Horová , I. J. Leitch , L. Mucina , E. Pacini , L. Tichý , et al. 2014 Ecological and evolutionary significance of genomic GC content diversity in monocots. Proceedings of the National Academy of Sciences USA 111: E4096–E4102. 10.1073/pnas.1321152111 PMC4191780 25225383

[aps311254-bib-0062] Solís‐Lemus, C. , M. Yang , and C. Ané . 2016 Inconsistency of species tree methods under gene flow. Systematic Biology 65: 843–851. 27151419 10.1093/sysbio/syw030

[aps311254-bib-0063] Soltis, P. S. , and D. E. Soltis . 2009 The role of hybridization in plant speciation. Annual Review of Plant Biology 60: 561–588. 10.1146/annurev.arplant.043008.092039 19575590

[aps311254-bib-0064] Soto Gomez, M. , L. Pokorny , M. B. Kantar , F. Forest , I. J. Leitch , B. Gravendeel , P. Wilkin , et al. 2019 Data from: A customized target enrichment approach for developing a phylogenomic baseline for *Dioscorea* yams (Dioscoreaceae). Dryad Digital Repository. 10.5061/dryad.4n2fk97. PMC6580989 31236313

[aps311254-bib-0065] Stamatakis, A. 2014 RAxML version 8: A tool for phylogenetic analysis and post‐analysis of large phylogenies. Bioinformatics 30: 1312–1313. 24451623 10.1093/bioinformatics/btu033 PMC3998144

[aps311254-bib-0066] Terauchi, R. , V. A. Chikaleke , G. Thottappilly , and S. K. Hahn . 1992 Origin and phylogeny of Guinea yams as revealed by RFLP analysis of chloroplast DNA and nuclear ribosomal DNA. Theoretical and Applied Genetics 83: 743–751. 24202749 10.1007/BF00226693

[aps311254-bib-0067] Vaidya, G. , D. J. Lohman , and R. Meier . 2011 SequenceMatrix: Concatenation software for the fast assembly of multi‐gene datasets with character set and codon information. Cladistics 27: 171–180. 10.1111/j.1096-0031.2010.00329.x 34875773

[aps311254-bib-0068] Vatanparast, M. , A. Powell , J. J. Doyle , and A. N. Egan . 2018 Targeting legume loci: A comparison of three methods for target enrichment bait design in Leguminosae phylogenomics. Applications in Plant Sciences 6: e1036. 29732266 10.1002/aps3.1036 PMC5895186

[aps311254-bib-0069] Villaverde, T. , L. Pokorny , S. Olsson , M. Rincón‐Barrado , M. G. Johnson , E. M. Gardner , N. J. Wickett , et al. 2018 Bridging the micro‐ and macroevolutionary levels in phylogenomics: Hyb‐Seq solves relationships from populations to species and above. New Phytologist 220: 636–650. 30016546 10.1111/nph.15312

[aps311254-bib-0070] Vincent, H. , J. Wiersema , S. Kell , H. Fielder , S. Dobbie , N. P. Castañeda‐Álvarez , L. Guarino , et al. 2013 A prioritized crop wild relative inventory to help underpin global food security. Biological Conservation 167: 265–275.

[aps311254-bib-0071] Viruel, J. , J. G. Segarra‐Moragues , L. Raz , F. Forest , P. Wilkin , I. Sanmartín , and P. Catalán . 2016 Late Cretaceous‐Early Eocene origin of yams (*Dioscorea*, Dioscoreaceae) in the Laurasian Palaearctic and their subsequent Oligocene‐Miocene diversification. Journal of Biogeography 43: 750–762.

[aps311254-bib-0072] Viruel, J. , F. Forest , O. Paun , M. W. Chase , D. Devey , R. Sousa Couto , J. G. Segarra‐Moragues , et al. 2018 A nuclear *Xdh* analysis of yams (*Dioscorea*, Dioscoreaceae) congruent with plastid trees reveals a new Neotropical lineage. Botanical Journal of the Linnean Society 187: 232–246.

[aps311254-bib-0073] Wang, X. , D. Chen , Y. Wang , and J. Xie . 2015 De novo transcriptome assembly and the putative biosynthetic pathway of steroidal sapogenins of *Dioscorea composita* . PLoS ONE 10: e0124560. 25860891 10.1371/journal.pone.0124560 PMC4393236

[aps311254-bib-0074] Weitemier, K. , S. C. K. Straub , R. C. Cronn , M. Fishbein , R. Schmickl , A. McDonnell , and A. Liston . 2014 Hyb‐Seq: Combining target enrichment and genome skimming for plant phylogenomics. Applications in Plant Sciences 2: 1400042. 10.3732/apps.1400042 PMC4162667 25225629

[aps311254-bib-0075] Wickett, N. J. , S. Mirarab , N. Nguyen , T. Warnow , E. Carpenter , N. Matasci , S. Ayyampalayam , et al. 2014 Phylotranscriptomic analysis of the origin and early diversification of land plants. Proceedings of the National Academy of Sciences USA 111: E4859–E4868. 10.1073/pnas.1323926111 PMC4234587 25355905

[aps311254-bib-0076] Wilkin, P. , P. Schols , M. W. Chase , K. Chayamarit , C. A. Furness , S. Huysmans , F. Rakotonasolo , et al. 2005 A plastid gene phylogeny of the yam genus, *Dioscorea*: Roots, fruits and Madagascar. Systematic Botany 30: 736–749.

[aps311254-bib-0077] Wolf, P. G. , T. A. Robison , M. G. Johnson , M. A. Sundue , W. L. Testo , and C. J. Rothfels . 2018 Target sequence capture of nuclear‐encoded genes for phylogenetic analysis in ferns. Applications in Plant Sciences 6: e1148. 10.1002/aps3.1148 PMC5991577 30131890

[aps311254-bib-0078] Wu, Z. G. , W. Jiang , N. Mantri , X. Q. Bao , S. L. Chen , and Z. M. Tao . 2015 Transciptome analysis reveals flavonoid biosynthesis regulation and simple sequence repeats in yam (*Dioscorea alata* L.) tubers. BMC Genomics 16: 346. 25924983 10.1186/s12864-015-1547-8 PMC4415240

[aps311254-bib-0079] Yang, Z. 2006 Computational molecular evolution. Oxford University Press, Oxford, United Kingdom.

[aps311254-bib-0080] Zhang, L. , B. Cao , and C. Bai . 2013 New reports of nuclear DNA content for 66 traditional Chinese medicinal plant taxa in China. Caryologia 66: 375–383.

[aps311254-bib-0081] Zhang, C. , M. Rabiee , E. Sayyari , and S. Mirarab . 2018 ASTRAL‐III: Polynomial time species tree reconstruction from partially resolved gene trees. BMC Bioinformatics 19: 153. 29745866 10.1186/s12859-018-2129-y PMC5998893

