# Supplementary material for: A customized nuclear target enrichment approach for developing a phylogenomic baseline for Dioscorea yams (Dioscoreaceae)
Source: Appl Plant Sci. 2019 Jun 13;7(6):e11254. doi: 10.1002/aps3.11254 (PMC6580989; doi:10.1002/aps3.11254)
Supplement: Supplementary file 1 — APPENDIX S1. Specimen source information for the 25 taxa enriched using Dioscorea‐specific baits, and for the four newly generated transcriptomes (the latter are indicated with an asterisk). [file APS3-7-e11254-s001.docx]

**APPENDIX S1.** Specimen source information for the 25 taxa enriched using *Dioscorea-*specific baits, and for the four newly generated transcriptomes (the latter are indicated with an asterisk).**^a^**

| **Species** | **Voucher [Collector number, herbarium]** | **NCBI Sequence Read Archive (SRA) accession** |
| --- | --- | --- |
| ***Dioscorea*** |  |  |
| *Dioscorea antaly* | Wilkin et al. 1104, K | SAMN11290802 |
| *Dioscorea birmanica* | Thapyai 409, BKF | SAMN11290804 |
| *Dioscorea calcicola* | Wilkin 814, K | SAMN11290794 |
| *Dioscorea campestris* | Wood 9178, K | SAMN11290814 |
| *Dioscorea caucasica* | no data, N/A (Kew living collection 1980-2270) | SAMN11290818 |
| *Dioscorea cochleariapiculata** | Soto, M. & Viruel J. 002, K | SAMN11290800 |
| *Dioscorea communis* | no data, K | SAMN11290809 |
| *Dioscorea communis** | Soto, M. & Viruel J. Cit08, K | SAMN11290810 |
| *Dioscorea cordata* | F Axelrod 8407, UPR | SAMN11290803 |
| *Dioscorea daunea* | Thapyai 518, BKF | SAMN11290805 |
| *Dioscorea glandulosa* | JRI Wood et al., 18973, K | SAMN11290812 |
| *Dioscorea hombuka* | Caddick et al. 329, K | SAMN11290797 |
| *Dioscorea kituiensis* | G Mwachala et al. 949a, EA | SAMN11290808 |
| *Dioscorea mayottensis* | Hladik 8507, P | SAMN11290796 |
| *Dioscorea membranacea* | MW Chase 21050, K | SAMN11290819 |
| *Dioscorea minima* | O Tellez 13086, no data | SAMN11290817 |
| *Dioscorea* x *monandra* | Wood et al., 22679, no data | SAMN11290813 |
| *Dioscorea nitens* | Thapyai 452, BKF | SAMN11290806 |
| *Dioscorea nummularia* | RJ Johns et al. 9824, K | SAMN11290795 |
| *Dioscorea orthogoneura* | JRI Wood & E Guzman 17511, K | SAMN11290816 |
| *Dioscorea pentaphylla* | MW Chase 21045, K | SAMN11290801 |
| *Dioscorea pohlii* | LCS Assis & MK Ladeira 67, K | SAMN11290815 |
| *Dioscorea prazeri* | Wilkin et al. 1075, K | SAMN11290820 |
| *Dioscorea rockii* | MW Chase 21052, K | SAMN11290821 |
| *Dioscorea sansibarensis* | Hladik 6764, P | SAMN11290799 |
| *Dioscorea soso** | Soto, M. & Viruel J. 001, K | SAMN11290798 |
| *Dioscorea sylvatica** | Soto, M. & Viruel J. 003, K | SAMN11290807 |
| *Dioscorea tentaculigera* | Thapyai 436, BKF | SAMN11290811 |
| ***Tacca*** |  |  |
| *Tacca chantrieri* | G. Lim 1A, BH | SAMN11290823 |
| ***Trichopus*** |  |  |
| *Trichopus zeylanicus* | M Chase & M Fay 16354, K | SAMN11290822 |

^a^See main text for data access for the remaining taxa. Herbarium abbreviations follow Index Herbariorum (Thiers, B. 2017 [Continuously updated]. Index Herbariorum, part 1: The herbaria of the world. New York Botanical Garden, Bronx, New York, USA. Website http://sweetgum.nybg.org/ih/ [accessed 10 June 2018].). We obtained DNA for *D. caucasica* from the Kew DNA bank (ID: 35312), and there is no voucher information available for it.
