# Supplementary material for: A customized nuclear target enrichment approach for developing a phylogenomic baseline for Dioscorea yams (Dioscoreaceae)
Source: Appl Plant Sci. 2019 Jun 13;7(6):e11254. doi: 10.1002/aps3.11254 (PMC6580989; doi:10.1002/aps3.11254)
Supplement: Supplementary file 2 — APPENDIX S2. Supplementary methods for detecting and filtering potential paralogs. [file APS3-7-e11254-s002.docx]

**APPENDIX S2.** Supplementary methods for detecting and filtering potential paralogs.

HybPiper produced paralog warnings for 26 loci, indicating that for one or more taxa it detected multiple contigs that spanned over 85% of the reference gene sequence (potential paralogs are indicated in the gene characterization file in the Dryad Digital Repository [<https://doi.org/10.5061/dryad.4n2fk97>; Soto Gomez et al., 2019]. For each of these instances, we inferred additional gene trees that used all of the identified gene copies per taxon, with potential paralogs retrieved using the paralog_retriever.py script in HybPiper. In 22 of these cases, we detected what we believe are ancient paralogs, recent paralogs, or allelic variants (nucleotide identity 26–97%); alignment inspection clearly pointed to nine of these gene copies as representing “chimeras” of orthologous and paralogous sequences that may result from PCR amplification of pre- and post-enriched libraries, Illumina sequencing, or retrieval artifacts in HybPiper. We filtered out all putative paralogous, allelic, and chimeric sequences to leave the single most similar copy (65–99% nucleotide identity), which we interpreted as the sequence most likely to be orthologous to other included taxa, and retained it for analysis. The resulting topologies (after alignment trimming; data not shown) were consistent with our current understanding of Dioscoreales phylogeny (Wilkin et al., 2005; Viruel et al., 2016, 2018). However, the topology of five additional gene trees was in one or more ways substantially divergent from our understanding of relationships in Dioscoreales, indicative of additional possible paralogy issues. One of these cases, gene 168, was not identified by HybPiper as a possible paralogy instance, but was instead picked up by visual inspection of the other 233 gene trees. For all five instances, it appears that HybPiper preferentially pulled out one of two copies per taxon, and so presumably HybPiper was unable to recover both copies during the initial analysis because one may have been too short or lacked sufficient read depth (M. G. Johnson, Texas Tech University, personal communication). We interpreted these five cases as being the result of a single early gene duplication and separated the resulting two distinct clades (also visible in alignments) into two paralogous genes (each with only a partial taxon set), increasing the total number of recovered genes by five total. We used the resulting two paralogs of each of these five genes as alternate user-provided low- to single-copy nuclear gene references in HybPiper to search for these sequences in additional taxa. We recovered each gene copy from 20–30 taxa and ran the HybPiper script paralog_retriever.py on these. Again, we used sequence similarity and tree-based methods to retain the copy most likely to be orthologous to other sampled taxa.

**LITERATURE CITED**

Soto Gomez, M., L. Pokorny, M. B. Kantar, F. Forest, I. J. Leitch, B. Gravendeel, P. Wilkin, et al. 2019. Data from: A customized target enrichment approach for developing a phylogenomic baseline for *Dioscorea* yams (Dioscoreaceae). Dryad Digital Repository. https://doi.org/10.5061/dryad.4n2fk97

Viruel, J., J. G. Segarra-Moragues, L. Raz, F. Forest, P. Wilkin, I. Sanmartín, and P. Catalán. 2016. Late Cretaceous-Early Eocene origin of yams (*Dioscorea*, Dioscoreaceae) in the Laurasian Palaearctic and their subsequent Oligocene-Miocene diversification. *Journal of Biogeography* 43: 750–762.

Viruel, J., F. Forest, O. Paun, M. W. Chase, D. Devey, R. Sousa Couto, J. G. Segarra-Moragues, et al. 2018. A nuclear *Xdh* analysis of yams (*Dioscorea*, Dioscoreaceae) congruent with plastid trees reveals a new Neotropical lineage. *Botanical Journal of the Linnean Society* 187: 232-246.

Wilkin, P., P. Schols, M. W. Chase, K. Chayamarit, C. A. Furness, S. Huysmans, F. Rakotonasolo, et al. 2005. A plastid gene phylogeny of the yam genus, *Dioscorea*: Roots, fruits and Madagascar. *Systematic Botany* 30: 736-749.
