# Supplementary material for: A customized nuclear target enrichment approach for developing a phylogenomic baseline for Dioscorea yams (Dioscoreaceae)
Source: Appl Plant Sci. 2019 Jun 13;7(6):e11254. doi: 10.1002/aps3.11254 (PMC6580989; doi:10.1002/aps3.11254)

APPENDIX S3. Phylogenetic relationships in *Dioscorea* inferred from partitioned maximum likelihood analyses on a concatenated matrix of 264 genes recovered using target enrichment with the *Dioscorea*-specific baits designed here. The data matrix was partitioned by gene (genes with similar models and model parameters were pooled) and analyzed using corresponding DNA substitution models. Values next to branches are bootstrap support values; thick lines represent 100% bootstrap support. Lineages in red are major crops; blue labels indicate previously identified crop wild relatives. Scale bar indicates estimated substitutions per site.

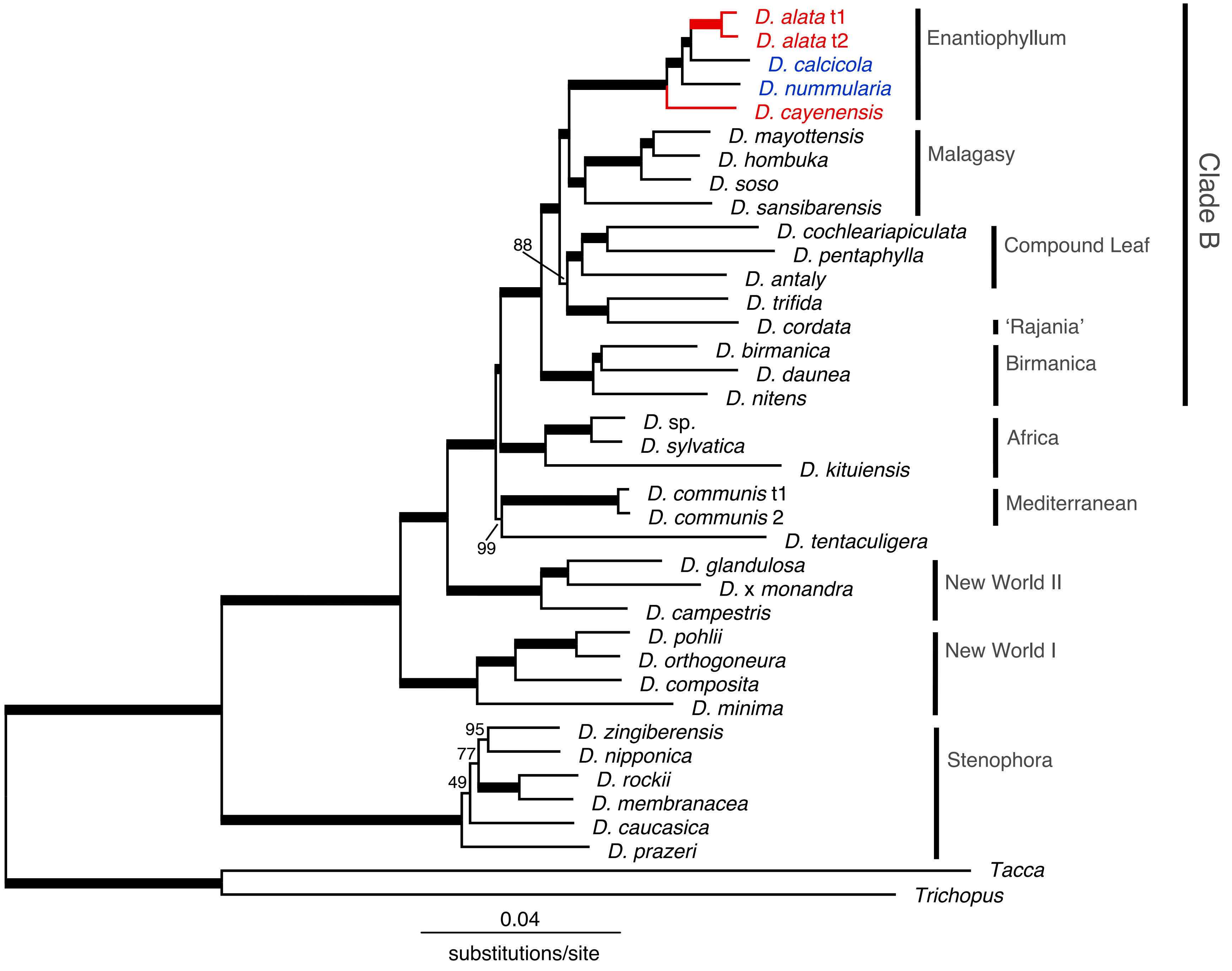

Supplement: Supplementary file 3 — APPENDIX S3. Phylogenetic relationships in Dioscorea inferred from partitioned maximum likelihood analyses on a concatenated matrix of 264 genes recovered using target enrichment with the Dioscorea‐specific baits designed here. [file APS3-7-e11254-s003.pdf]
