# Supplementary material for: A customized nuclear target enrichment approach for developing a phylogenomic baseline for Dioscorea yams (Dioscoreaceae)
Source: Appl Plant Sci. 2019 Jun 13;7(6):e11254. doi: 10.1002/aps3.11254 (PMC6580989; doi:10.1002/aps3.11254)

APPENDIX S4. Phylogenetic relationships in *Dioscorea* inferred from unpartitioned maximum likelihood analyses on a concatenated matrix of 264 genes recovered using target enrichment with the *Dioscorea*-specific baits designed here. Values next to branches are bootstrap support values; thick lines represent 100% bootstrap support. Lineages in red are major crops; blue labels indicate previously identified crop wild relatives. Scale bar indicates estimated substitutions per site.

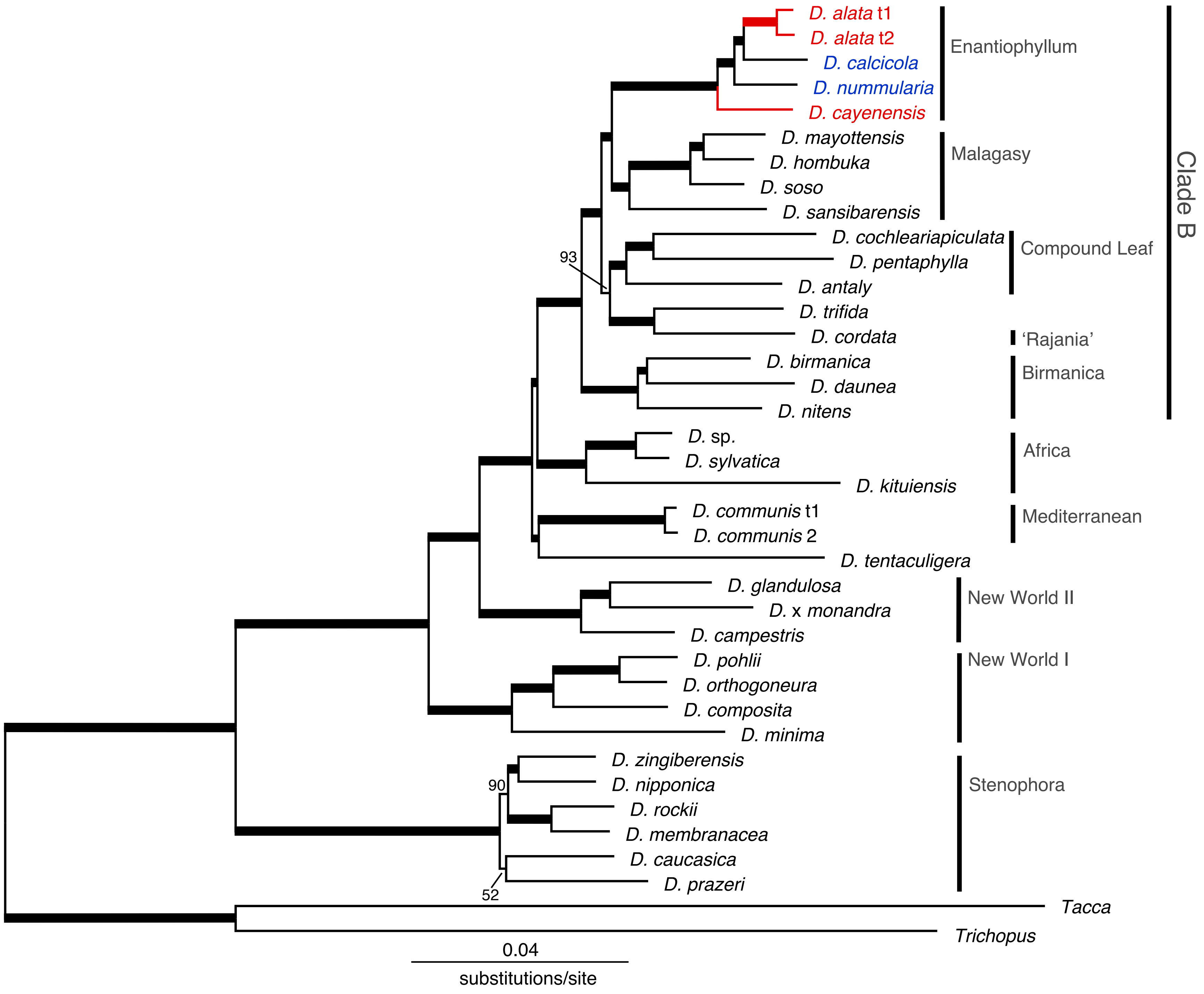

Supplement: Supplementary file 4 — APPENDIX S4. Phylogenetic relationships in Dioscorea inferred from unpartitioned maximum likelihood analyses on a concatenated matrix of 264 genes recovered using target enrichment with the Dioscorea‐specific baits designed here. [file APS3-7-e11254-s004.pdf]
