# Supplementary material for: A customized nuclear target enrichment approach for developing a phylogenomic baseline for Dioscorea yams (Dioscoreaceae)
Source: Appl Plant Sci. 2019 Jun 13;7(6):e11254. doi: 10.1002/aps3.11254 (PMC6580989; doi:10.1002/aps3.11254)

APPENDIX S5. Phylogenetic relationships in *Dioscorea* inferred from coalescent-based analyses of a partial 232 gene set that excluded 32 potential paralogs. Genes were recovered using target enrichment with the *Dioscorea*-specific baits designed here. Values next to branches are local posterior probabilities (LPP) and multilocus bootstrap support (MLB), respectively; thick branches have 1.0 LPP and 100% bootstrap support. Lineages in red are major crops; blue labels indicate previously identified crop wild relatives. Scale bar shows coalescent units for internal branches (not estimated by ASTRAL for terminal branches)

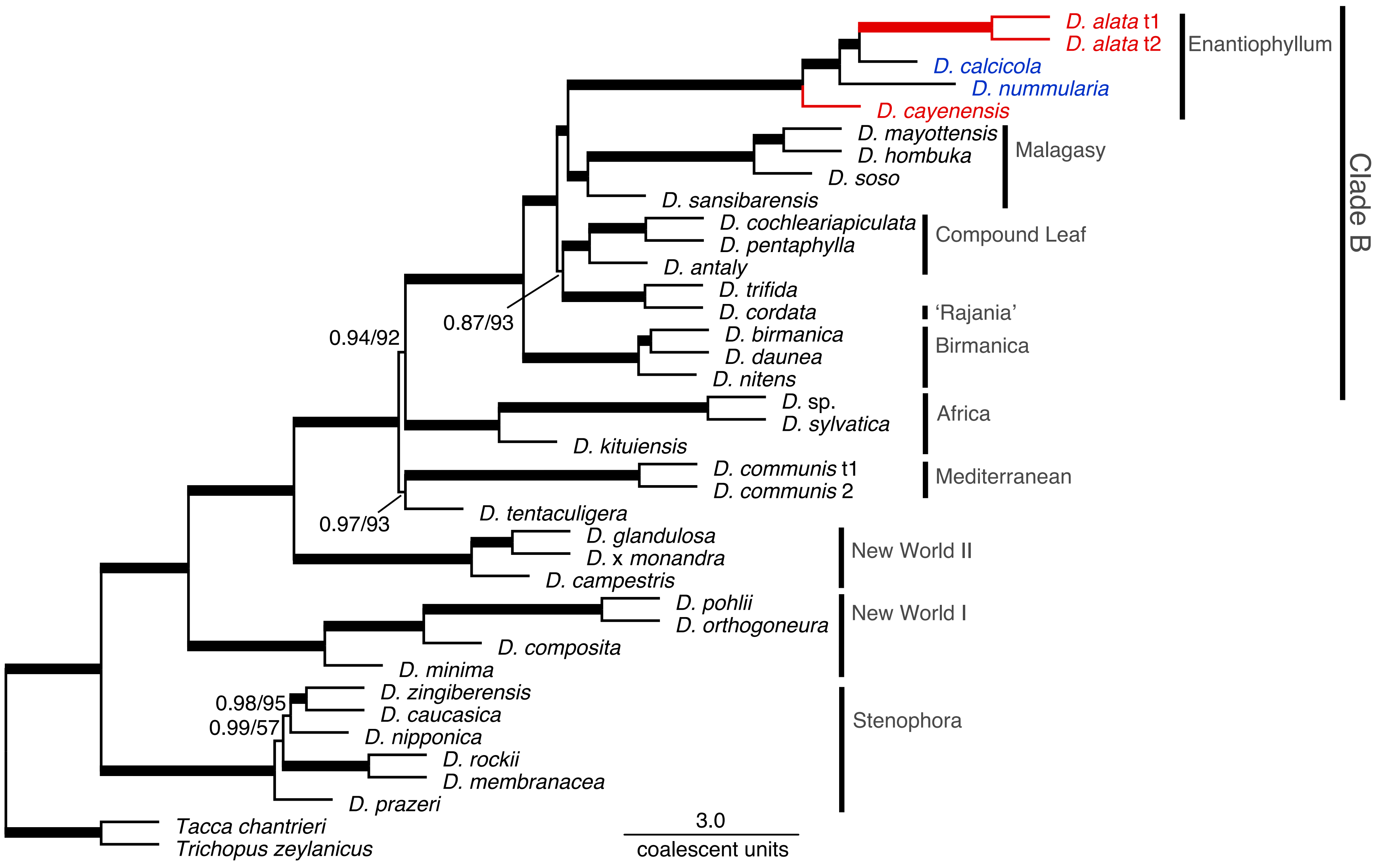

Supplement: Supplementary file 5 — APPENDIX S5. Phylogenetic relationships in Dioscorea inferred from coalescent‐based analyses of a partial 232 gene set that excluded 32 potential paralogs. [file APS3-7-e11254-s005.pdf]
